# Salvianolic acid B: a promising cardioprotective agent

**DOI:** 10.3389/fimmu.2025.1681358

**Published:** 2025-11-21

**Authors:** Yingchun Shao, Li Zhai, Shan Jiang, Fusheng Sun, Xuedong Liu

**Affiliations:** Qingdao Hospital, University of Health and Rehabilitation Sciences (Qingdao Municipal Hospital), Qingdao, China

**Keywords:** polyphenols, salvianolic acid B, cardiovascular diseases, CVD, signaling pathways

## Abstract

*Salvia miltiorrhiza* Bunge [Lamiaceae; Salviae miltiorrhizae radix et rhizoma] is a core herb in traditional Chinese medicine widely used for treating cardiovascular diseases (CVD). Its key bioactive compound, salvianolic acid B (SalB), has emerged as a promising therapeutic agent for CVD. Modern pharmacological studies have demonstrated that SalB exerts comprehensive cardioprotective effects through multiple mechanisms, including antioxidant and anti-inflammatory activities, induction of mitochondrial autophagy, enhancement of endothelial function, anti-fibrotic actions, and improvement of hemorheology. These properties underscore its significant value in both the prevention and treatment of CVD. However, current research on SalB in the context of CVD remains relatively fragmented. To address this gap, this review systematically consolidates existing research findings on SalB in the cardiovascular field, providing an in-depth analysis of its sources, pharmacological mechanisms, efficacy characteristics, compatibility strategies, and dosage form optimization. Furthermore, by integrating both preclinical and clinical data, this review comprehensively evaluates the safety and efficacy of SalB, aiming to offer theoretical support and clear research directions to facilitate the substantive transformation of this traditional bioactive compound into a modern CVD therapeutic drug.

## 1 Introduction

*Salvia miltiorrhiza* Bunge [Lamiaceae; Salviae miltiorrhizae radix et rhizoma] is widely used in clinical practice for its therapeutic effects, including coronary artery dilation, anti-myocardial ischemia, microcirculatory improvement, reduction of myocardial oxygen consumption, enhancement of cardiac function, and modulation of blood pressure, lipid levels, anticoagulation, and thrombosis, along with liver protection and immune regulation (1–3). Salvianolic acid B (SalB), the primary active compound in *S. miltiorrhiza*, has shown significant potential in the prevention and treatment of cardiovascular diseases (CVD) owing to its diverse pharmacological properties.

SalB demonstrates a broad spectrum of biological activities, including antioxidant, anti-inflammatory, anti-thrombotic, anti-apoptotic, anti-aging, and vascular and mitochondrial protection (4–6), establishing it as a central focus in CVD research. Numerous studies have emphasized SalB’s ability to neutralize reactive oxygen species, thereby reducing oxidative stress-induced cellular damage (7–9), which is critical for CVD prevention. Moreover, SalB inhibits platelet aggregation and thrombosis (10–12), playing a key role in the prevention of acute cardiovascular events such as myocardial infarction and stroke. Its multi-target mechanism of action further supports its broad applicability in treating cardiovascular conditions. Additionally, its natural origin and low toxicity make SalB a promising candidate for long-term adjuvant therapy, positioning it as a potential cardioprotective agent.

Currently, *S. miltiorrhiza* injection and salvianolate are extensively used in clinical practice for the treatment of coronary heart disease, angina, cerebral infarction, and related liver, kidney, and lung disorders, with SalB as the primary active component. However, a monomeric preparation of SalB has yet to be developed, limiting the full utilization of its medicinal potential. This review systematically examines the sources, limitations, and pharmacological mechanisms of SalB, providing a comprehensive summary of recent advancements in CVD research, drug compatibility, and the latest developments in formulation design and clinical studies. The review aims to provide theoretical support and practical guidance for the clinical translation of SalB, addressing existing research fragmentation and promoting its transformation into a modern cardiovascular therapeutic agent. Furthermore, the in-depth analysis of SalB’s pharmacological characteristics and mechanisms of action presented here serves as a valuable template for future pharmaceutical chemistry research, paving the way for the development of novel structural drugs with enhanced efficacy, reduced toxicity, and improved pharmacokinetic properties.

## 2 The source and limitations of SalB

SalB (C_36_H_30_O_16_) is a monomeric compound extracted from the dried roots of *S. miltiorrhiza* Bunge (13–15). As one of the most significant water-soluble components of *S. miltiorrhiza*, SalB is distinguished by its high concentration and potent bioactivity (16–18). Maximizing the extraction of SalB from *S. miltiorrhiza* is therefore critical for enhancing its medicinal value. Current methods for SalB separation and purification include water extraction, ethanol precipitation, macroporous resin adsorption, ultrasonic extraction, high-speed countercurrent chromatography, subcritical water extraction, and supercritical CO_2_ extraction (19–21). However, the molecular structure of SalB, formed by the condensation of three molecules of danshensu and one molecule of caffeic acid, contains ester bonds and a five-membered ring, which makes it prone to ester hydrolysis and ring-opening reactions (22–24). Moreover, while the parent compound of SalB can be quantified in systemic circulation following oral administration, its absolute bioavailability is severely limited by factors such as intestinal instability, poor membrane permeability, and susceptibility to structural degradation, all of which restrict its systemic exposure (25–27). These challenges pose safety risks in clinical settings and hinder the use of SalB in oral liquid formulations. Therefore, to address the challenge of its low oral bioavailability, several strategies can be employed. First, modifications to dosage formsecati as nanoparticles, injectable hydrogels, and core-shell nanofibersles, enhance SalB’s stability and delivery efficiency. Additionally, structural modifications to SalB itself represent an effective means to optimize its pharmacokinetic properties and improve bioavailability. Moreover, selecting high-quality *S. miltiorrhiza* varieties, can increase SalB extraction yields. Simultaneously, the entire extraction processionus raw material selection to extraction, purification, and storageation,y be systematically optimized, incorporating advanced technologies like supercritical CO2 extraction and subcritical water extraction. These innovations have significantly enhanced the therapeutic efficacy of SalB and paved the way for future drug development.

## 3 The pharmacological action pathway of SalB

**Figure 1:**
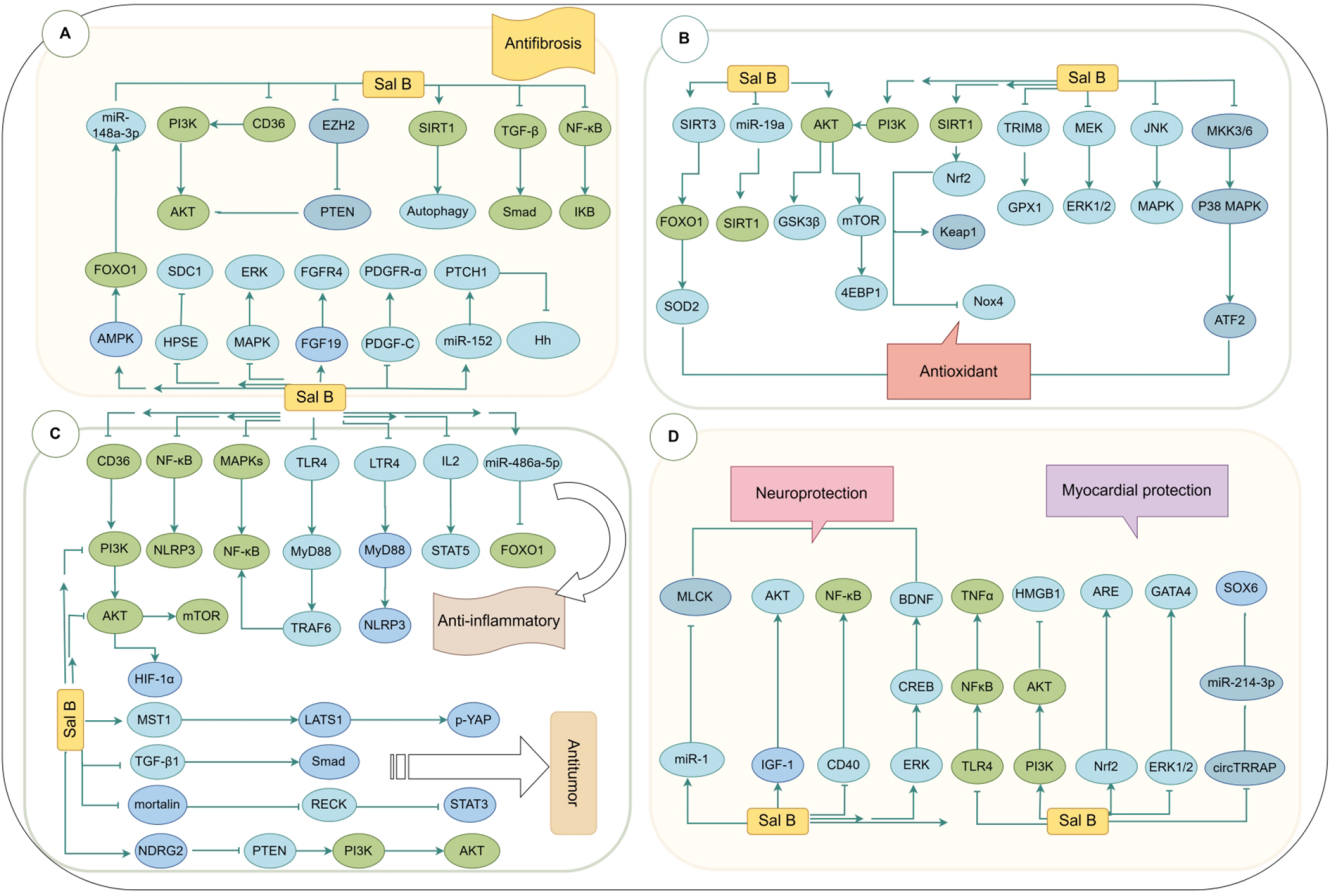
The pharmacological action pathway of SalB. **(A)** The mechanism of SalB in inhibiting fibrosis. **(B)** The mechanism by which SalB alleviates ROS-induced injury. **(C)** The mechanism of SalB’s anti-inflammatory and anti-tumor effects. **(D)** The mechanism by which SalB regulates neuronal function and myocardial injury.

SalB is a multi-target natural compound with remarkable pharmacological activity. By modulating various signaling pathways, it exerts biological effects such as anti-oxidation, anti-inflammation, anti-fibrosis, anti-cancer, cardiovascular protection, and neuroprotection. For instance, SalB regulates TGF-β1/Smad (28), MAPK/ERK (28), NF-κB/IκB (29), miR-152/PTCH1/Hh (30), CD36/PI3K/AKT (31), FGF19/FGFR4 (32), EZH2/PTEN/AKT (33), SIRT1-autophagy (34), PDGF-C/PDGFR-α (35), AMPK/FOXO1/miR-148a-3p (36), and HPSE/SDC1 (37) signaling pathways to exert anti-fibrotic effects (**Figure 1A**). It also regulates TRIM8/GPX1 (38), AKT/GSK3β (39), Nrf2/Keap1 (40), Nrf2/Nox4 (41), PI3K/AKT (42), JNK/MAPK (43), AKT/mTOR/4EBP1 (44), MKK3/6-p38MAPK/ATF2 (44), MEK/ERK1/2 (45), miR-19a/SIRT1 (46), and SIRT3/FOXO1/SOD2 (47) signaling pathways for antioxidant effects (**Figure 1B**) and modulates NF-κB/NLRP3 (48), LTR4/MyD88/NLRP3 (49), MAPKs/NF-κB (50), CD36/PI3K/AKT (31), IL2/STAT5 (51), TLR4/MyD88/TRAF6 (52), miR-486a-5p/FOXO1 (53), and AKT/mTOR (54) signaling pathways to exert anti-inflammatory effects (**Figure 1C**). In addition, SalB regulates AKT/mTOR (55), Hippo/YAP (56), PI3K/AKT/HIF-1α (57), TGF-β1/Smad (58), mortalin/RECK/STAT3 (59), and NDRG2/PTEN/PI3K/AKT (60) signaling pathways to promote anti-cancer effects (**Figure 1C**) and exerts neuroprotective effects by modulating CD40/NF-κB (61), IGF-1/AKT (62), ERK/CREB/BDNF (63), and miRNA-1/MLCK (64) signaling pathways (**Figure 1D**). Furthermore, SalB confers myocardial protection by regulating circTRRAP/miR-214-3p/SOX6 (65), ERK1/2/GATA4 (66), Nrf2/ARE (67), PI3K/AKT/HMGB1 (68), and TLR4/NF-κ-B/TNF-α (69) signaling pathways (**Figure 1D**). Notably, the pharmacological effects of SalB are not isolated but interact through intricate signaling pathways in various pathological states, forming an extensive regulatory network. For example, SalB exerts anti-fibrotic effects via the CD36/PI3K/AKT pathway while simultaneously exhibiting antioxidant, anti-inflammatory, and anti-cancer effects through the AKT/mTOR pathway, thereby establishing a multi-layered regulatory mechanism. Moreover, SalB synergistically inhibits oxidative stress and inflammation through the Nrf2/NLRP3 and NF-κB/NLRP3 pathways. This multi-pathway synergy significantly enhances SalB’s therapeutic potential, offering a unique advantage in treating complex diseases.

## 4 The therapeutic effect of SalB on CVD

Various preparations derived from *S. miltiorrhiza* are currently widely used in clinical practice, including *S. miltiorrhiza* injection, oral liquids, tablets, compound granules, and *S. miltiorrhiza*-ligustrazine injection, all of which primarily contain active components from *S. miltiorrhiza*. Additionally, the *S. miltiorrhiza* extract tanshinone IIA has been developed into a monomeric preparation—sodium tanshinone IIA sulfonate injection—used clinically as an adjunct therapy for coronary heart disease, angina pectoris, and myocardial infarction (MI). Furthermore, preparations containing salvianolic acids, such as salvianolic acids for injection and salvianolate for injection, are employed to promote blood circulation and alleviate collateral obstruction, treating conditions like mild to moderate cerebral infarction and stable angina pectoris in coronary heart disease. However, there are currently no clinical applications for SalB, a primary monomer component of salvianolic acids, in the treatment of cardiovascular and cerebrovascular diseases, with related research still largely in the basic research phase.

### 4.1 Atherosclerosis

Atherosclerosis is closely linked to vascular endothelial dysfunction and inflammation (70–72). SalB alleviates inflammation and thrombosis by inhibiting the activation of the TNF-α-induced NF-κB/NLRP3 pathway and platelet activation (48, 73–75). Additionally, SalB protects blood vessels by reducing lipid accumulation in the vessel walls and inhibiting VEGF-induced vascular hyperpermeability, which prevents the uptake of 125I-LDL and thereby slows the progression of atherosclerosis (76–78). Furthermore, SalB safeguards endothelial cells by inhibiting arginase activity and the activation of Piezo1 (79, 80). It also prevents NLRP3 inflammasome activation and pyroptosis triggered by endoplasmic reticulum (ER) stress through the suppression of the AMPK/FOXO4/KLF4 and Syndecan-4/Rac1/ATF2 signaling pathways, effectively protecting endothelial progenitor cells from damage (81). Additionally, in LPS-induced human aortic smooth muscle cells and ApoE^-/-^ mice, SalB inhibited the expression of COX-2, MMP-2, and MMP-9 both *in vitro* and *in vivo* (82), suggesting that SalB may slow the progression of atherosclerosis through its anti-inflammatory effects and by decreasing vascular matrix degradation. LDL oxidation exacerbates inflammation within the vessel wall and promotes foam cell formation, accelerating atherosclerosis. As an antioxidant, SalB inhibits LDL oxidation and reduces the associated inflammatory response by blocking JAK/STAT1 activation induced by IFN-γ (83, 84). Furthermore, CD36, a high-affinity receptor for oxidized LDL (ox-LDL), is widely expressed in various cell types and plays a critical role in lipid metabolism, inflammation, and atherosclerosis (85–87). Previous studies have demonstrated that SalB effectively inhibits the binding of CD36 to its ligand, thereby mitigating its detrimental effects in atherosclerosis and inflammation (88, 89).

**Figure 2:**
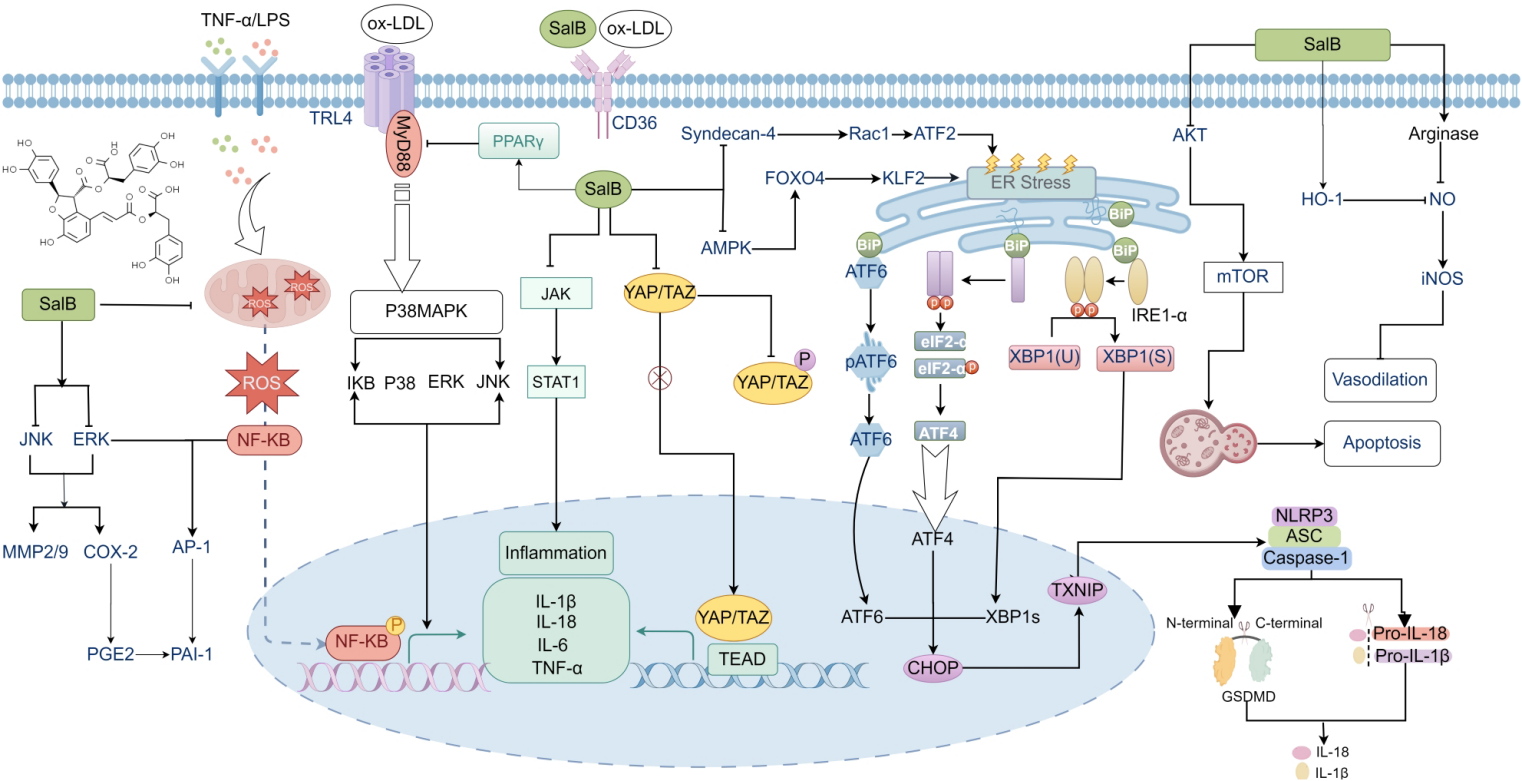
The protective mechanism of SalB against atherosclerosis (AS). SalB ameliorates AS by modulating multiple signaling pathways, including ERK/JNK/MMP-9/2, ERK/JNK/COX-2/PGE2, NF-κB/ERK/AP-1/PAI-1, PPARγ/TLR4/MyD88/p38MAPK, JAK/STAT1, YAP/TAZ/JNK, Syndecan-4/Rac1/ATF2, AMPK/FOXO4/KLF2, AKT/mTOR, and HO-1/NO/iNOS axes.

SalB also exerts regulatory effects on dendritic cells, macrophages, and pericytes in the context of atherosclerosis. Additionally, SalB interferes with the maturation process of dendritic cells by activating PPARγ, thus reducing their ability to stimulate immune responses (90). It also effectively inhibits inflammation and autophagy dysfunction in LDLR^-/-^ mice and RAW264.7 cells by regulating the MAPKs/NF-κB and AKT/mTOR signaling pathways, reducing the progression of atherosclerosis (50, 91). Additionally, by inhibiting the YAP/TAZ/JNK signaling pathway, SalB reduces the abnormal proliferation of pericytes and the release of inflammatory factors, offering a novel intervention strategy for preventing and treating atherosclerosis (92). The protective mechanism of SalB on atherosclerosis is summarized in **Figure 2**.

In summary, SalB effectively mitigates multiple pathological aspects of atherosclerosis by modulating mechanisms in endothelial cells, macrophages, dendritic cells, pericytes, and immune responses. Its ability to inhibit inflammation, reduce lipid deposition, prevent vascular fibrosis, and enhance endothelial function provides a strong scientific foundation for its potential as a therapeutic agent in the treatment of atherosclerosis.

### 4.2 MI

**Figure 3:**
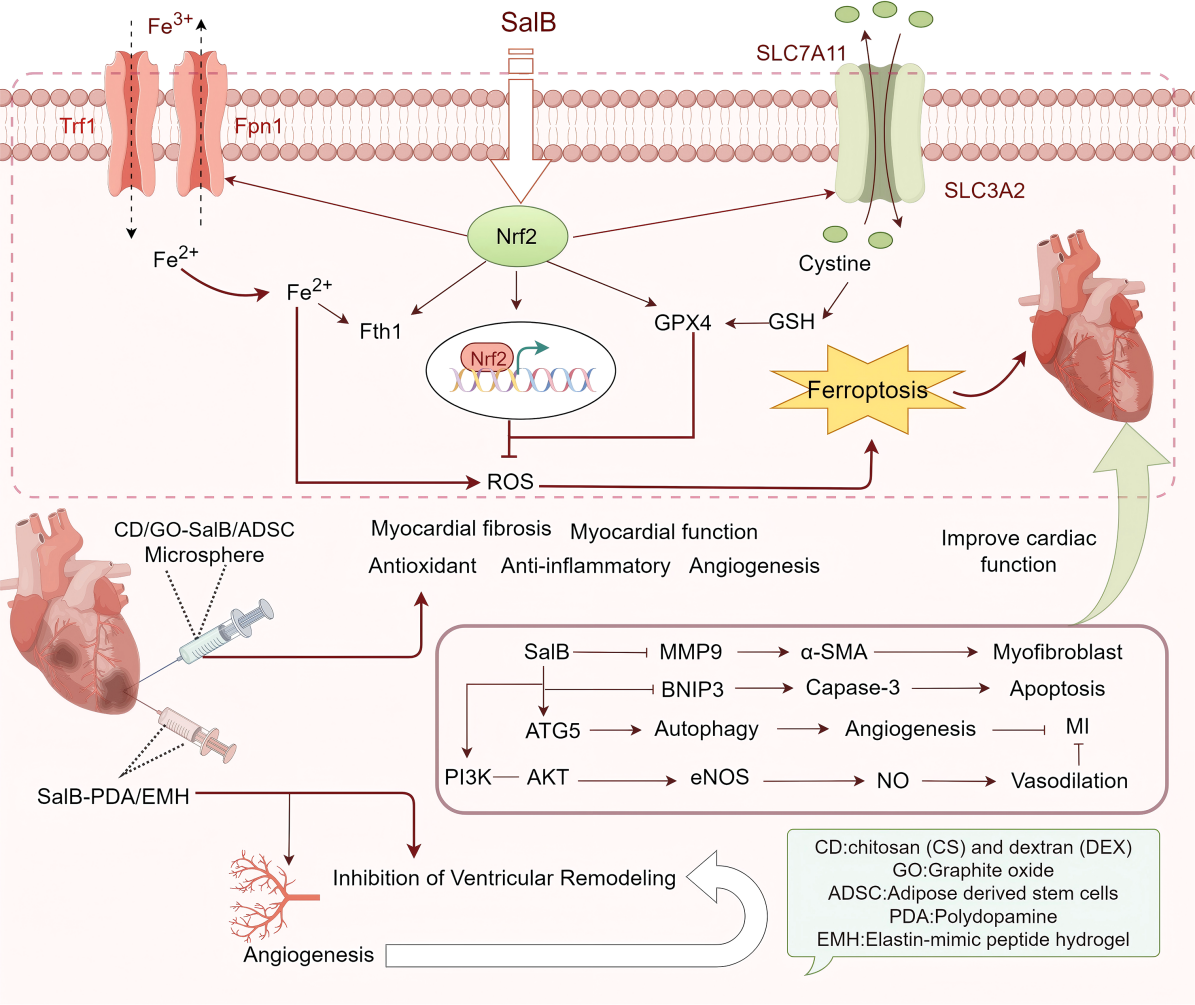
Protective mechanism of SalB against myocardial infarction (MI). It can play a significant myocardial protective role in MI by regulating Nrf2/ferroptosis, MMP9/α-SMA, BNIP3/caspase3, PI3K/AKT, ATG5/autophagy signaling pathways.

In 2008, He et al. first reported that SalB exhibited myocardial protection in mice with MI, though these findings were later retracted (93). However, research into SalB’s protective effects against MI continued, with significant advancements in subsequent years. In 2009, Tan et al. demonstrated that pre-treatment of endogenous precursor cells (EPCs) with SalB, followed by transplantation alongside bone marrow mesenchymal stem cells (BMSCs) into the ischemic myocardium, resulted in notable improvements, suggesting SalB’s protective role in MI (94). In 2010, Jiang et al. identified SalB as a competitive inhibitor of matrix metalloproteinase-9 (MMP-9), which effectively reduced ischemic myocardial fibrosis, further supporting its myocardial protective effects (95). Zhao et al. (2012) confirmed Tan et al.’s findings, showing that SalB promoted the expansion of EPCs, improved the ischemic microenvironment, and enhanced BMSC survival and differentiation into cardiomyocytes (96). In 2014, Han et al. discovered that SalB’s protective effect in MI was linked to the promotion of mesenchymal stem cell (MSC) differentiation into endothelial cells, rather than cardiomyocytes (97). These studies collectively suggest that SalB’s myocardial protection is associated with the differentiation of EPCs, BMSCs, and MSCs. Further research has elucidated the mechanisms underlying SalB’s protection against MI, including its role in promoting nitric oxide (NO) production, autophagy, angiogenesis, and inhibiting the NLRP3 inflammasome, apoptosis, and ferroptosis (98–103). The compatibility of traditional Chinese medicines has also demonstrated therapeutic advantages in clinical settings, with early studies exploring SalB’s potential in MI treatment. For example, Deng et al. found that combining SalB with ginsenoside Rg1 in a 2:5 ratio improved cardiac contractility in MI rats (104). To address the challenge of SalB’s low bioavailability in MI treatment, various drug carriers have been developed for more effective delivery. Qiu et al. created lipid-polymer mixed nanoparticles (LPNs) for co-delivery of SalB and panax notoginseng saponins (PNS), modified with arginyl-glycyl-aspartic acid (RGD) to form RGD-S/P-LPNs nanoparticles (105). These nanoparticles exhibited excellent serum stability and sustained drug release, significantly enhancing the therapeutic effect on MI *in vivo* (105). Shoba et al. developed a core-shell nanofiber system designed for phased delivery, releasing SalB from the core and magnesium L-ascorbic acid 2-phosphate (MAAP) from the shell, providing an effective vector for MI treatment (106). Chen et al. designed an elastin-mimicking peptide hydrogel (EMH) for delivering SalB-loaded dopamine nanoparticles (SalB-PDA), forming a SalB-PDA/EMH injectable peptide hydrogel with self-healing and slow-release properties (107). This hydrogel has been shown to inhibit ventricular remodeling and promote angiogenesis, offering a novel approach to MI treatment (107). These innovative drug carriers not only enhance SalB’s efficacy but also open new possibilities for the treatment of MI and related diseases. In summary, the core mechanisms of SalB’s protection in MI primarily involve modulating stem cell behavior, enhancing angiogenesis, and suppressing maladaptive inflammatory responses and cell death pathways. The above content is summarized in **Figure 3**.

### 4.3 Myocardial ischemia-reperfusion injury (MI/RI)

**Figure 4:**
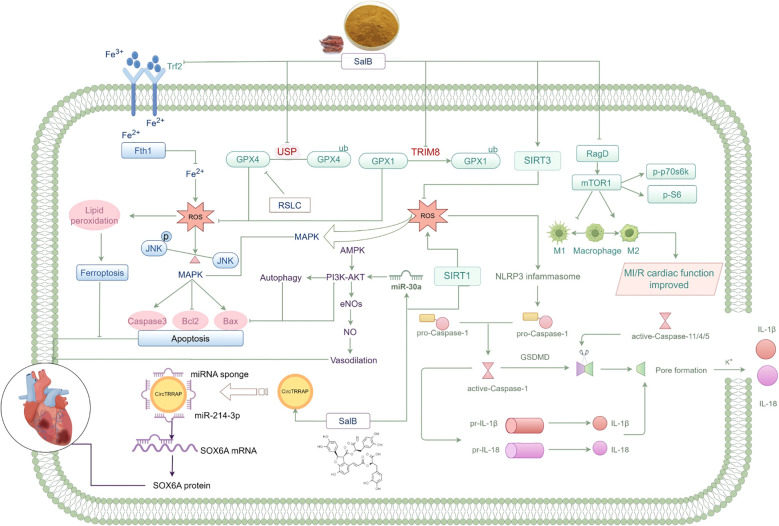
Protective mechanism of SalB on myocardial ischemia/reperfusion (MI/RI) injury. SalB confers cardioprotection against MI/RI by modulating the GPX4/ROS/ferroptosis, TRIM8/GPX1, SIRT3/ROS/NLRP3 inflammasome, SIRT1/ROS/MAPK, RagD/mTOR1, AMPK/PI3K/AKT/eNOS/NO, miR-30a/PI3K/AKT, and circTRRAP/miR-214-3p/SOX6A signaling axes, thereby attenuating oxidative stress, inflammation, apoptosis, and autophagic dysfunction while preserving myocardial function.

In 2011, Qiao et al. investigated the effects of the ethanol extract of *S. miltiorrhiza* Bunge on MI/RI and found that SalB, a key component of the extract, exhibited protective effects, particularly in preventing MI/R-induced oxidative damage in the myocardium (108). Subsequent studies by Qiao et al. and Gao et al. further confirmed SalB’s myocardial protective effect in MI/RI, showing significant improvements in cardiac function and tissue integrity (109, 110). Further investigations revealed that SalB’s protective mechanisms in MI/R were linked to enhancing cardiac contractility, scavenging reactive oxygen species, reducing inflammation, lipid peroxidation, apoptosis, and ferroptosis, and inhibiting autophagy (111–113). Key regulatory mechanisms involved the activation of the PI3K/AKT and SIRT1/MAPK pathways, alongside inhibition of the circTRRAP/miR-214-3p/SOX6 and TRIM8/GPX1 axes (38, 65, 68, 114) (**Figure 4**). Additionally, Deng et al. demonstrated that combining SalB with ginsenoside Rg1 in a 2:5 ratio could protect against MI and improve MI/RI outcomes (104, 115). Overall, the core mechanisms by which SalB protects against MI/RI—focused on antioxidation, anti-inflammation, and regulation of various cell death pathways—overlap with its effects in MI, underscoring its broad role in counteracting ischemic damage. However, SalB’s role in MI/RI appears to emphasize mitigating reperfusion-specific injuries, such as the acute oxidative burst, distinguishing it from its action in MI alone.

### 4.4 Cardiac hypertrophy

Studies on SalB’s therapeutic effects in cardiac hypertrophy remain limited, but some important findings have emerged. Liu et al. demonstrated that SalB effectively blocked the hypertrophic response in neonatal rat cardiomyocytes exposed to angiotensin II (116). The anti-hypertrophic effect of SalB was linked to its inhibition of poly (ADP-ribose) polymerase-1 (PARP-1), preventing NAD^+^ depletion in cells (116). This inhibition occurred through SalB’s suppression of the antioxidant functions of NOX2 and NOX4 (116). Further research by Yu et al. using a transverse aortic constriction (TAC)-induced cardiac hypertrophy model revealed that SalB provided superior protection against myocardial damage compared to metoprolol (66). Moreover, Ma et al. suggested that SalB may offer advantages over carvedilol in treating left ventricular hypertrophy, as SalB inhibits both the ERK signaling pathway and the β-adrenergic receptor, whereas carvedilol acts solely as a β-receptor blocker (117). These findings highlight SalB’s potential as a novel therapeutic agent for cardiac hypertrophy.

### 4.5 Diabetic cardiomyopathy (DCM)

DCM, a condition commonly associated with diabetes mellitus, is characterized by structural and functional abnormalities in the myocardium (118–120). SalB exerts significant protective effects against DCM. Specifically, Li et al. found that SalB alleviates diabetic myocardial fibrosis by inhibiting insulin-like growth factor binding protein 3 (IGFBP3) and promoting angiogenesis (121). Furthermore, Luo et al. demonstrated that SalB inhibits the TGF-β1 signaling pathway by upregulating Smad7 expression, which reduces myocardial fibrosis and inflammatory cell infiltration, effectively alleviating diabetic myocardial damage (122). In summary, SalB exhibits substantial therapeutic potential in mitigating myocardial pathological damage caused by diabetes through multiple mechanisms, including anti-fibrosis, anti-inflammation, and pro-angiogenesis, supporting its use in treating DCM.

### 4.6 Septic cardiomyopathy (SCM)

SCM is a systemic inflammatory response triggered by infection that impairs heart function (123–125). Due to its potent anti-inflammatory and antioxidant properties, SalB has shown protective effects against myocardial damage caused by sepsis. Chen et al. found that SalB enhances the mitochondrial unfolded protein response (UPRmt) by activating transcription factor 5 (ATF5), restoring protein folding balance in mitochondria and alleviating mitochondrial damage induced by sepsis (126). The core mechanism of SalB in SCM revolves around mitochondrial protection through ATF5-mediated UPRmt activation, a pathway distinct from its roles in fibrosis inhibition or stem cell regulation observed in conditions like MI, cardiac hypertrophy, or DCM. While SalB’s anti-inflammatory and antioxidant effects are common across various cardiac pathologies, its specific engagement with mitochondrial quality control in SCM underscores its capacity to address disease-specific mechanisms driven by severe infection and metabolic stress. This mechanism forms a theoretical foundation for the clinical application of SalB in treating SCM, offering new approaches for managing this condition.

### 4.7 Uremic cardiomyopathy (UC)

UC, a heart disease associated with renal failure, is often characterized by myocardial fibrosis, declining cardiac function, and elevated oxidative stress (127–129). Ma et al. developed a rat model of UC to study the effects of SalB on cardiac function, ventricular hypertrophy, myocardial fibrosis, and inflammatory markers at various time points (130). Their findings indicated that SalB treatment improved cardiac function, reduced myocardial fibrosis, and alleviated inflammation in UC rats, thereby delaying disease progression. The cardioprotective effect of SalB in UC primarily results from its anti-fibrotic and anti-inflammatory properties, mechanisms that align with its actions in other cardiovascular conditions, such as DCM and cardiac hypertrophy.

### 4.8 Doxorubicin-induced cardiomyopathy (DIC)

Doxorubicin (DOX), a widely used chemotherapeutic agent, is known for its cardiotoxicity, which induces oxidative stress, inflammation, and ER stress in cardiomyocytes, ultimately leading to cardiomyocyte apoptosis and heart injury (131–133). SalB mitigates ER stress by activating the PI3K/AKT signaling pathway, thereby inhibiting DOX-induced cardiomyocyte apoptosis (134). Additionally, SalB pretreatment prevents calcium overload and ER stress caused by DOX, with its protective mechanism involving the inhibition of TRP channel subfamily members 3 (TRPC3) and 6 (TRPC6) expression (135). These findings confirm that SalB exerts its protective effects against DOX-induced cardiotoxicity primarily by regulating ER stress and calcium homeostasis, highlighting its potential clinical value in treating DIC.

### 4.9 Cisplatin-induced cardiac injury

Cisplatin, a widely used chemotherapeutic agent, is commonly linked to severe cardiotoxicity and oxidative stress, resulting in cardiac dysfunction (136–138). SalB effectively mitigates cisplatin-induced cardiac damage and oxidative stress, with its protective effect closely associated with the activation of the Nrf2 signaling pathway (67, 139). These findings establish Nrf2-mediated antioxidant pathway activation as the core mechanism through which SalB combats cisplatin-induced cardiotoxicity. This redox balance modulation differentiates its action from the calcium homeostasis regulation observed in DIC or the anti-fibrotic effects observed in DCM, highlighting SalB’s ability to selectively target specific pathological triggers.

## 5 Research on the compatibility and dosage forms of SalB

**Figure 5:**
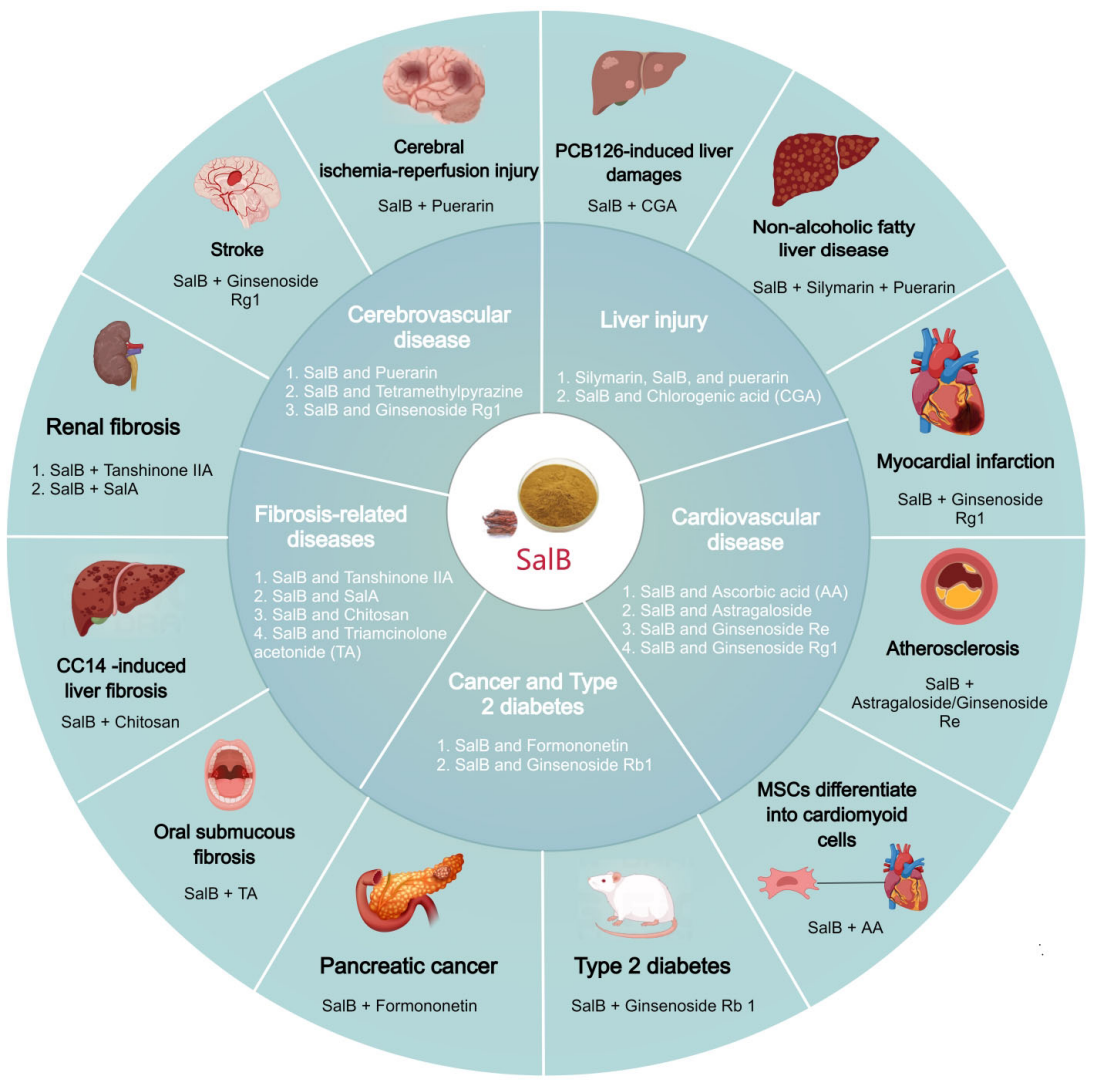
The combination therapy of SalB. SalB can be combined with ascorbic acid, astragaloside IV, ginsenoside Re, ginsenoside Rg1, puerarin, and tetramethylpyrazine for cardiocerebrovascular diseases; with puerarin and chlorogenic acid for hepatic injury; with tanshinone IIA, SalA, chitosan, and triamcinolone acetonide for fibrosis-related disorders; with formononetin for cancer; and with ginsenoside Rb1 for type 2 diabetes.

To overcome the limitations of SalB, numerous strategies, including drug compatibility and dosage form modifications, have been explored. In CVD, combining SalB with ascorbic acid has been shown to enhance the differentiation of MSCs into cardiomyocyte-like cells mediated by valproic acid and 5-azacytidine (140). This combination offers a promising approach for stem cell-based cardiac regeneration therapy. Moreover, combining SalB with astragaloside IV or ginsenoside Re significantly reduces arterial plaque area and lipid deposition (141, 142). Furthermore, the combination of SalB and ginsenoside Rg1 provides notable protection for cardiac function in patients with subacute MI, and it synergistically improves stroke treatment outcomes (143–145). Importantly, studies have demonstrated that this combined regimen does not cause any abnormal changes in the tissue structure of the brain, heart, kidneys, liver, or lungs within seven days (146), ensuring the safety of the combination and providing strong support for its clinical application. Beyond CVD, the combined drug strategy of SalB has also been explored in treating cerebrovascular diseases, cancer, liver damage, kidney disease, and oral mucosal fibrosis (**Figure 5**).

In terms of dosage form research, innovative drug carrier systems have been developed in recent years to improve the oral bioavailability, targeted delivery, stability, and efficacy of SalB. Nanoparticles, as a key drug delivery platform, can enhance the therapeutic effects of SalB by improving stability, targeting, and absorption efficiency. For instance, Zhang et al. employed methoxypolyethylene glycol-chitosan (PCS)-derived nanoparticles to load SalB, enhancing its oral delivery capability and kidney-targeted distribution (147). Catechol-modified chitosan nanocarriers can also assemble SalB through coordination, enabling its targeted delivery to the kidneys and enhancing its anti-renal fibrosis effects (148). Moreover, the nanoparticle system developed by Grossi et al., capable of crossing the blood-brain barrier, efficiently delivers SalB to the central nervous system, improving its neuroprotective effects (149).

Liposomes have also been demonstrated to be effective as SalB drug delivery systems. Isacchi et al. developed SalB-loaded liposomes, which significantly improved the bioavailability and prolonged the pharmacodynamic effects of SalB (150), offering a new therapeutic option for treating neuropathic pain. Additionally, Shi et al. encapsulated SalB in liposomes modified with the cell-penetrating peptide TAT to enhance its skin repair potential (151).

In the development of composite scaffolds, Qin et al. used 3D bioprinting technology to create a SalB-sodium alginate-gelatin composite porous scaffold, which was applied to diabetic wounds. This scaffold was found to accelerate wound healing significantly (152). Similarly, Li et al. loaded SalB into chitosan microspheres, evenly distributing them in three dimensions and fixing them on the surface of porous hydroxyapatite (HA) scaffolds, thus creating a new type of bone tissue engineering scaffold (153). This composite scaffold demonstrated significant effects in promoting cell proliferation, adhesion, and differentiation, highlighting its potential for bone tissue regeneration. Additionally, polylactic acid (PLA) and graphene oxide (GO) have been utilized as composite scaffolds for SalB, enabling sustained release of SalB to promote bone tissue regeneration (154).

Based on the drug-loading capabilities of hydrogels, Chen et al. developed a SalB-polydopamine nanoparticle/elastin-mimic peptide hydrogel with suitable mechanical strength and self-healing properties (107). This hydrogel facilitates myocardial tissue repair and regeneration by enabling the long-term release of SalB directly to the infarct area. In another approach, injectable hydrogels composed of hyaluronic acid and gelatin were designed to deliver SalB and vascular endothelial growth factor, promoting synergistic brain tissue repair (155).

Bioactive glass scaffolds have also been explored for SalB delivery. Wu et al. loaded SalB into a mesoporous bioactive glass scaffold (MBG) via physical adsorption, forming a SalB-MBG scaffold. This scaffold effectively sustained SalB release and significantly promoted new bone formation and angiogenesis in bone defect sites (156). Kan et al. developed SalB microporous osmotic pump controlled-release pellets (157), which exhibited excellent *in vitro* drug release performance and favorable *in vivo* pharmacokinetic properties, making them a promising option for treating CVD.

In conclusion, nanoparticles and liposomes improve the delivery efficiency and therapeutic efficacy of SalB by enhancing drug stability, targeting, and absorption. Composite scaffolds and hydrogels offer unique advantages for tissue repair and regeneration, thanks to their customizable and sustainable release properties. Additionally, innovative carrier materials, such as mesoporous bioactive glass scaffolds and controlled-release pellets, have expanded the potential applications of SalB, particularly in regenerative medicine and the treatment of CVD.

## 6 Preclinical and clinical research status

Preclinical studies have shown that SalB provides significant protective effects across multiple organs, including the heart, liver (158–160), brain (161–163), kidneys (164–166), and lungs (167–169), indicating its broad therapeutic potential for treating cardiovascular, liver, renal, neurodegenerative, and pulmonary diseases. Notably, SalB’s cardiovascular protective effects are strongly supported by extensive preclinical animal data, with some indications advancing to clinical trials (**Table 1**). For example, intravenous administration of SalB for angina pectoris and coronary artery disease has entered Phase I clinical trials (CTR20192236). Cheng et al. assessed the safety, tolerability, and pharmacokinetic profile of SalB in healthy subjects (170), revealing no serious adverse events across all dose groups, with favorable safety and tolerability (170), supporting its potential application in CVD. Additionally, SalB has shown promising results in other clinical settings. A study of 42 patients with oral submucous fibrosis reported that combining triamcinolone acetonide with SalB significantly improved mouth opening and reduced burning sensations, with no adverse effects observed (171). In another clinical trial involving 60 patients with chronic hepatitis B-related liver fibrosis, six months of oral SalB tablets significantly improved liver fibrosis markers without side effects (172). Furthermore, multiple patents covering SalB’s extraction methods and therapeutic strategies for CVD further validate its clinical and methodological value (**Table 2**). In conclusion, SalB holds substantial promise for clinical translation in cardiovascular therapeutics. With growing clinical evidence, it may become a safe and effective treatment option, opening new pathways for patient care.

**Table 1:** Preclinical and clinical trial studies on the cardiovascular therapeutic effects of SalB.

Research category	Drug	Disease	Mode of administration	Target	Reference
Preclinical study	SalB	Atrial fbrillation	Tail vein injection	AMPK	(36)
Preclinical study	SalB	MI/RI	Intravenous injection	TRIM8	(38)
Preclinical study	SalB	MI/RI	Intravenous injection	GPX4	(43)
Preclinical study	SalB	Atherosclerosis	Intraperitoneal injection	NF-κB	(48)
Preclinical study	SalB	Atherosclerosis	Intraperitoneal injection	MAPKs	(50)
Preclinical study	SalB	TAC	Gavage	ERK1/2	(66)
Preclinical study	SalB	MI/RI	Intraperitoneal injection	PI3K	(68)
Preclinical study	SalB	MI	Tail vein injection	MMP-9	(95)
Preclinical study	SalB-MSC	MI	Spot injection	VEGF	(97)
Preclinical study	SalB	MI	Intraperitoneal injection	AMPK	(98)
Preclinical study	SalB	AMI	Intravenous injection	/	(99)
Preclinical study	SalB	AMI	Tail vein injection	SIRT1	(102)
Preclinical study	SalB	MI	Intraperitoneal injection	Nrf2	(103)
Preclinical study	SalB+ginsenoside Rg1 (60 mg/kg)	AMI	Gavage	/	(104)
Preclinical study	RGD-S/P-LPNs	AMI	Intravenous injection	/	(105)
Preclinical study	SalB-PDA/pre-EMH	MI	Intramuscular injection	/	(107)
Preclinical study	SalB	MI/RI	Gavage	/	(109)
Preclinical study	SalB	MI/RI	Perfusion	/	(110)
Preclinical study	SalB	MI/RI	Intravenous administration	/	(111)
Preclinical study	SalB	MI/RI	Gavage	RagD	(112)
Preclinical study	SalB+ginsenoside Rg1 (10 mg/kg)	MI/RI	Reperfusion time	/	(115)
Preclinical study	SalB	Diabetic cardiomyopathy	Intraperitoneal injection	IGFBP3	(121)
Preclinical study	SalB	Diabetic cardiomyopathy	Gavage	Smad7	(122)
Preclinical study	SalB	Doxorubicin (DOX)-induced cardiotoxicity	Intravenous injection	TRPC3/6	(135)
Preclinical study	SalB	Septic cardiomyopathy	Intraperitoneal injection	ATF5	(126)
Preclinical study	SalB	Uremic cardiomyopathy	Intraperitoneal injection	/	(130)
Preclinical study	SalB	Cisplatin-induced cardiac injury	Gavage	Nrf2	(139)
Preclinical study	SalB	Atherosclerosis	Intraperitoneal injection	Piezo1	(80)
Preclinical study	SalB	Atherosclerosis	Per os	/	(83)
Preclinical study	SalB+Astragaloside IV	Atherosclerosis	Intraperitoneal injection	/	(141)
Preclinical study	SalB+ginsenoside Rg1 (30 mg/kg)	MI	Intraperitoneal injection	MMP-9	(143)
Preclinical study	SMND-309	MI	Tail vein injection	Bcl-2	(173)
Preclinical study	CD/GO-SalB/ADSC	AMI	Intramuscular injection	CD31	(174)
Preclinical study	SalB	MI/RI	Intravenous administration	SIRT3	(175)
Preclinical study	SalB	Hypertension	Gavage	AT1	(176)
Preclinical study	SalB	Portal hypertension	Perfusion	iNOS	(177)
Preclinical study	SalB	Portal Pressure	Gavage	RhoA	(178)
Preclinical study	SalB	Atherosclerosis	Per os	MMP-2/9	(179)
Phase I clinical trial	SalB	Angina pectoris; Coronary artery disease	Intravenous injection	/	CTR20192236

**Table 2:** The relevant patents of SalB in cardiovascular diseases.

Patent name	Publication number	Application	Publication date
Application of SalB in the preparation of protective and synergistic antitumor drugs for cardiotoxicity induced by arsenic trioxide	CN103230390B	Cardiotoxicity	2016-01-13
A time-delay controlled-release tablet for the treatment of coronary heart disease and its preparation method	CN109453132A	Coronary heart disease	2019-03-12
A drug composition for the treatment of myocardial injury in Kawasaki disease and its application	CN111617088A	Kawasaki disease myocardial injury	2020.09.04
SalB-supported 3D printing degrades intravascular stents	CN114470344A	Injured vessel	2022-05-13
SalB complex and its preparation and application	CN113082014B	Ischemic heart disease	2022-05-17
Application of SalB in the preparation of drugs for myocardial protection in patients with myocardial infarction during perioperative PCI	CN115998727A	Myocardial infarction	2023-04-25
Application of SalB in the preparation of drugs for diseases with lymphatic hypogenesis	CN117462529A	Dilated cardiomyopathy	2024-01-30
A long-acting sustained-release salvianolic acid B injectable hydrogel for heart failure treatment and its preparation method	CN115554235B	Heart failure	2023-08-25
Nickel-titanium alloy surface chitosan-SalB coating, preparation method and application thereof	CN117379602B	Thrombus	2024-03-15
SalB or total saponin of notoginseng and its application	CN117100733B	Lung cancer heart disease	2024-04-05
SalB is used to prepare drugs for the prevention and treatment of cardiotoxic-related diseases caused by PD-1/PD-L1 targeted inhibitors	CN118021786A	Cardiotoxicity	2024-05-14

## 7 Conclusions and prospects

SalB, a polyphenolic compound extracted from *S. miltiorrhiza* Bunge, exhibits remarkable cardiovascular protective potential. Its multi-target mechanism includes a range of biological effects such as antioxidant, anti-inflammatory, anti-fibrotic, anti-thrombotic, and anti-apoptotic activities, along with promoting angiogenesis, making it highly applicable for treating CVD such as MI, MI/R injury, DCM, drug-induced cardiomyopathy, and atherosclerosis. Notably, SalB monomer preparations (injection) have entered clinical trials for CVD, marking a shift from an “active ingredient” in traditional Chinese medicine formulations to a modern “single chemical drug,” signifying a critical advancement in the modernization of traditional Chinese medicine. Therefore, SalB is well-positioned to become a key clinical treatment for CVD. Given this potential, future research should focus on several key areas: In basic research, efforts should be directed toward developing personalized treatment strategies based on SalB’s multi-target mechanisms for various cardiovascular conditions. In applied research, optimizing drug carrier design and formulating new dosage forms is essential to improve SalB’s stability, bioavailability, and clinical efficacy, with the aim of advancing SalB monomer preparations toward market release. In clinical translation, large-scale, multicenter trials are necessary to validate SalB’s safety and efficacy while exploring potential combination therapies with other agents. In conclusion, with continued progress in basic research, drug development, and clinical translation, SalB—an agent with multiple mechanisms of action targeting diverse pathological processes—has the potential to become a mainstream clinical drug for CVD in the form of monomer preparations.

## References

[1] Zhang MX Huang XY Song Y Xu WL Li YL Li C . Astragalus propinquus schischkin and Salvia miltiorrhiza bunge promote angiogenesis to treat myocardial ischemia via Ang-1/Tie-2/FAK pathway. Front Pharmacol. (2022) 13:1103557. doi: 10.3389/fphar.2022.1103557, PMID: 36699092 PMC9868545

[2] Zhang GX Zhang YY Zhang XX Wang PQ Liu J Liu Q . Different network pharmacology mechanisms of Danshen-based Fangjis in the treatment of stable angina. Acta Pharmacol Sin. (2018) 39:952–60. doi: 10.1038/aps.2017.191, PMID: 29417948 PMC6256275

[3] Yuan T Chen Y Zhou X Lin X Zhang Q . Effectiveness and safety of Danshen injection on heart failure: Protocol for a systematic review and meta-analysis. Med (Baltimore). (2019) 98:e15636. doi: 10.1097/MD.0000000000015636, PMID: 31145280 PMC6709174

[4] Zhao M Mu F Lin R Gao K Zhang W Tao X . Chinese medicine-derived salvianolic acid B for disease therapy: A scientometric study. Am J Chin Med. (2024) 52:1359–96. doi: 10.1142/S0192415X2450054X, PMID: 39212495

[5] Sun JM Liu YX Liu YD Ho CK Tsai YT Wen DS . Salvianolic acid B protects against UVB-induced skin aging via activation of NRF2. Phytomedicine. (2024) 130:155676. doi: 10.1016/j.phymed.2024.155676, PMID: 38820663

[6] Xiang J Zhang C Di T Chen L Zhao W Wei L . Salvianolic acid B alleviates diabetic endothelial and mitochondrial dysfunction by down-regulating apoptosis and mitophagy of endothelial cells. Bioengineered. (2022) 13:3486–502. doi: 10.1080/21655979.2022.2026552, PMID: 35068334 PMC8974099

[7] Liu J Zhang Y Qu D Zhang H Wang L Lau CW . Salvianolic acid B ameliorates vascular endothelial dysfunction through influencing a bone morphogenetic protein 4-ROS cycle in diabetic mice. Life Sci. (2021) 286:120039. doi: 10.1016/j.lfs.2021.120039, PMID: 34637797

[8] Yang MC You FL Wang Z Liu XN Wang YF . Salvianolic acid B improves the disruption of high glucose-mediated brain microvascular endothelial cells via the ROS/HIF-1alpha/VEGF and miR-200b/VEGF signaling pathways. Neurosci Lett. (2016) 630:233–40. doi: 10.1016/j.neulet.2016.08.005, PMID: 27497919

[9] Wang QQ Wang M Li Y Liu YH Sun LQ . Attenuation of oxidative stress-induced cell apoptosis and pyroptosis in RSC96 cells by salvianolic acid B. Chin J Integr Med. (2022) 28:243–8. doi: 10.1007/s11655-021-3507-2, PMID: 35084700

[10] Li M Zhao C Wong RN Goto S Wang Z Liao F . Inhibition of shear-induced platelet aggregation in rat by tetramethylpyrazine and salvianolic acid B. Clin Hemorheol Microcirc. (2004) 31:97–103., PMID: 15310944

[11] Neves MAD Ni TT Mackeigan DT Shoara AA Lei X Slavkovic S . Salvianolic acid B inhibits thrombosis and directly blocks the thrombin catalytic site. Res Pract Thromb Haemost. (2024) 8:102443. doi: 10.1016/j.rpth.2024.102443, PMID: 38993621 PMC11238050

[12] Liu L Li J Zhang Y Zhang S Ye J Wen Z . Salvianolic acid B inhibits platelets as a P2Y12 antagonist and PDE inhibitor: evidence from clinic to laboratory. Thromb Res. (2014) 134:866–76. doi: 10.1016/j.thromres.2014.07.019, PMID: 25077998

[13] Qin T Rasul A Sarfraz A Sarfraz I Hussain G Anwar H . Salvianolic acid A & B: potential cytotoxic polyphenols in battle against cancer via targeting multiple signaling pathways. Int J Biol Sci. (2019) 15:2256–64. doi: 10.7150/ijbs.37467, PMID: 31592132 PMC6775286

[14] Liang Q Liu X Peng X Luo T Su Y Xu X . Salvianolic acid B in fibrosis treatment: a comprehensive review. Front Pharmacol. (2024) 15:1442181. doi: 10.3389/fphar.2024.1442181, PMID: 39139645 PMC11319160

[15] Li J Li R Wu X Zheng C Shiu PH Rangsinth P . An update on the potential application of herbal medicine in promoting angiogenesis. Front Pharmacol. (2022) 13:928817. doi: 10.3389/fphar.2022.928817, PMID: 35928282 PMC9345329

[16] Guo SS Wang ZG . Salvianolic acid B from Salvia miltiorrhiza bunge: A potential antitumor agent. Front Pharmacol. (2022) 13:1042745. doi: 10.3389/fphar.2022.1042745, PMID: 36386172 PMC9640750

[17] Zhang Y Zhai W Fan M Wu J Wang C . Salvianolic Acid B Significantly Suppresses the Migration of Melanoma Cells via Direct Interaction with beta-Actin. Molecules. (2024) 29. doi: 10.3390/molecules29040906, PMID: 38398656 PMC10892080

[18] Tian LL Wang XJ Sun YN Li CR Xing YL Zhao HB . Salvianolic acid B, an antioxidant from Salvia miltiorrhiza, prevents 6-hydroxydopamine induced apoptosis in SH-SY5Y cells. Int J Biochem Cell Biol. (2008) 40:409–22. doi: 10.1016/j.biocel.2007.08.005, PMID: 17884684

[19] Kan S Li J Huang W Shao L Chen D . Microsphere resin chromatography combined with microbial biotransformation for the separation and purification of salvianolic acid B in aqueous extract of roots of Salvia multiorrihza Bunge. J Chromatogr A. (2009) 1216:3881–6. doi: 10.1016/j.chroma.2009.02.084, PMID: 19296955

[20] Li C Ma Y Xu Y Qiu R Shen X Huang L . Ultrasonic-assisted nanofiltration separation recovering salvianolic acid B from ethanol wastewater. Ultrason Sonochem. (2024) 108:106967. doi: 10.1016/j.ultsonch.2024.106967, PMID: 38917596 PMC11255954

[21] Zhang M Ignatova S Liang Q Wu Jun F Sutherland I Wang Y . Rapid and high-throughput purification of salvianolic acid B from Salvia miltiorrhiza Bunge by high-performance counter-current chromatography. J Chromatogr A. (2009) 1216:3869–73. doi: 10.1016/j.chroma.2009.02.067, PMID: 19281995

[22] Zhang X Zou L Li J Xu B Wu T Fan H . Salvianolic acid B and danshensu induce osteogenic differentiation of rat bone marrow stromal stem cells by upregulating the nitric oxide pathway. Exp Ther Med. (2017) 14:2779–88. doi: 10.3892/etm.2017.4914, PMID: 28966669 PMC5615234

[23] Lu P Xing Y Xue Z Ma Z Zhang B Peng H . Pharmacokinetics of salvianolic acid B, rosmarinic acid and Danshensu in rat after pulmonary administration of Salvia miltiorrhiza polyphenolic acid solution. BioMed Chromatogr. (2019) 33:e4561. doi: 10.1002/bmc.4561, PMID: 31017297

[24] Habtemariam S . Molecular pharmacology of rosmarinic and salvianolic acids: potential seeds for alzheimer's and vascular dementia drugs. Int J Mol Sci. (2018) 19:458. doi: 10.3390/ijms19020458, PMID: 29401682 PMC5855680

[25] He G Chen G Liu W Ye D Liu X Liang X . Salvianolic acid B: A review of pharmacological effects, safety, combination therapy, new dosage forms, and novel drug delivery routes. Pharmaceutics. (2023) 15:2235. doi: 10.3390/pharmaceutics15092235, PMID: 37765204 PMC10538146

[26] Wu YT Chen YF Hsieh YJ Jaw I Shiao MS Tsai TH . Bioavailability of salvianolic acid B in conscious and freely moving rats. Int J Pharm. (2006) 326:25–31. doi: 10.1016/j.ijpharm.2006.07.003, PMID: 16905282

[27] Gao DY Han LM Zhang LH Fang XL Wang JX . Bioavailability of salvianolic acid B and effect on blood viscosities after oral administration of salvianolic acids in beagle dogs. Arch Pharm Res. (2009) 32:773–9. doi: 10.1007/s12272-009-1517-2, PMID: 19471893

[28] Liu Q Lu J Lin J Tang Y Pu W Shi X . Salvianolic acid B attenuates experimental skin fibrosis of systemic sclerosis. BioMed Pharmacother. (2019) 110:546–53. doi: 10.1016/j.biopha.2018.12.016, PMID: 30530290

[29] Wang R Yu XY Guo ZY Wang YJ Wu Y Yuan YF . Inhibitory effects of salvianolic acid B on CCl(4)-induced hepatic fibrosis through regulating NF-kappaB/IkappaBalpha signaling. J Ethnopharmacol. (2012) 144:592–8. doi: 10.1016/j.jep.2012.09.048, PMID: 23041223

[30] Yu F Lu Z Chen B Wu X Dong P Zheng J . Salvianolic acid B-induced microRNA-152 inhibits liver fibrosis by attenuating DNMT1-mediated Patched1 methylation. J Cell Mol Med. (2015) 19:2617–32. doi: 10.1111/jcmm.12655, PMID: 26257392 PMC4627567

[31] Yan Y Zhou M Meng K Zhou C Jia X Li X . Salvianolic acid B attenuates inflammation and prevent pathologic fibrosis by inhibiting CD36-mediated activation of the PI3K-Akt signaling pathway in frozen shoulder. Front Pharmacol. (2023) 14:1230174. doi: 10.3389/fphar.2023.1230174, PMID: 37593175 PMC10427508

[32] Tian S Chen M Wang B Han Y Shang H Chen J . Salvianolic acid B blocks hepatic stellate cell activation via FGF19/FGFR4 signaling. Ann Hepatol. (2021) 20:100259. doi: 10.1016/j.aohep.2020.07.013, PMID: 32980439

[33] Lin P Qiu F Wu M Xu L Huang D Wang C . Salvianolic acid B attenuates tubulointerstitial fibrosis by inhibiting EZH2 to regulate the PTEN/Akt pathway. Pharm Biol. (2023) 61:23–9. doi: 10.1080/13880209.2022.2148169, PMID: 36524761 PMC9762854

[34] He Y Lu R Wu J Pang Y Li J Chen J . Salvianolic acid B attenuates epithelial-mesenchymal transition in renal fibrosis rats through activating Sirt1-mediated autophagy. BioMed Pharmacother. (2020) 128:110241. doi: 10.1016/j.biopha.2020.110241, PMID: 32450523

[35] Yao L Zhao R He S Feng Q Qiao Y Wang P . Effects of salvianolic acid A and salvianolic acid B in renal interstitial fibrosis via PDGF-C/PDGFR-alpha signaling pathway. Phytomedicine. (2022) 106:154414. doi: 10.1016/j.phymed.2022.154414, PMID: 36057144

[36] Liu J Sun Q Sun X Wang Q Zou G Wang D . Therapeutic effects of salvianolic acid B on angiotensin II-induced atrial fibrosis by regulating atrium metabolism via targeting AMPK/foxO1/miR-148a-3p axis. J Cardiovasc Transl Res. (2023) 16:341–57. doi: 10.1007/s12265-022-10303-3, PMID: 35984595 PMC10151312

[37] Hu Y Wang M Pan Y Li Q Xu L . Salvianolic acid B attenuates renal interstitial fibrosis by regulating the HPSE/SDC1 axis. Mol Med Rep. (2020) 22:1325–34. doi: 10.3892/mmr.2020.11229, PMID: 32626974 PMC7339410

[38] Lu B Li J Gui M Yao L Fan M Zhou X . Salvianolic acid B inhibits myocardial I/R-induced ROS generation and cell apoptosis by regulating the TRIM8/GPX1 pathway. Pharm Biol. (2022) 60:1458–68. doi: 10.1080/13880209.2022.2096644, PMID: 35968584 PMC9380432

[39] Wang D Lu X Wang E Shi L Ma C Tan X . Salvianolic acid B attenuates oxidative stress-induced injuries in enterocytes by activating Akt/GSK3beta signaling and preserving mitochondrial function. Eur J Pharmacol. (2021) 909:174408. doi: 10.1016/j.ejphar.2021.174408, PMID: 34364877

[40] Xiao Z Liu W Mu YP Zhang H Wang XN Zhao CQ . Pharmacological effects of salvianolic acid B against oxidative damage. Front Pharmacol. (2020) 11:572373. doi: 10.3389/fphar.2020.572373, PMID: 33343348 PMC7741185

[41] Liu B Cao B Zhang D Xiao N Chen H Li GQ . Salvianolic acid B protects against paraquat-induced pulmonary injury by mediating Nrf2/Nox4 redox balance and TGF-beta1/Smad3 signaling. Toxicol Appl Pharmacol. (2016) 309:111–20. doi: 10.1016/j.taap.2016.08.004, PMID: 27507327

[42] Wang M Sun GB Sun X Wang HW Meng XB Qin M . Cardioprotective effect of salvianolic acid B against arsenic trioxide-induced injury in cardiac H9c2 cells via the PI3K/Akt signal pathway. Toxicol Lett. (2013) 216:100–7. doi: 10.1016/j.toxlet.2012.11.023, PMID: 23201927

[43] Xu X Mao C Zhang C Zhang M Gong J Wang X . Salvianolic Acid B Inhibits Ferroptosis and Apoptosis during Myocardial Ischemia/Reperfusion Injury via Decreasing the Ubiquitin-Proteasome Degradation of GPX4 and the ROS-JNK/MAPK Pathways. Molecules. (2023) 28:4117. doi: 10.3390/molecules28104117, PMID: 37241859 PMC10224207

[44] Tang Y Jacobi A Vater C Zou X Stiehler M . Salvianolic acid B protects human endothelial progenitor cells against oxidative stress-mediated dysfunction by modulating Akt/mTOR/4EBP1, p38 MAPK/ATF2, and ERK1/2 signaling pathways. Biochem Pharmacol. (2014) 90:34–49. doi: 10.1016/j.bcp.2014.04.008, PMID: 24780446

[45] Lu B Ye Z Deng Y Wu H Feng J . MEK/ERK pathway mediates cytoprotection of salvianolic acid B against oxidative stress-induced apoptosis in rat bone marrow stem cells. Cell Biol Int. (2010) 34:1063–8. doi: 10.1042/CBI20090126, PMID: 20629637

[46] Guo Y Yang JH Cao SD Gao CX He Y Wang Y . Effect of main ingredients of Danhong Injection against oxidative stress induced autophagy injury via miR-19a/SIRT1 pathway in endothelial cells. Phytomedicine. (2021) 83:153480. doi: 10.1016/j.phymed.2021.153480, PMID: 33548866

[47] Wen F Zhang S Sun L Qian M Xu H . Salvianolic acid B inhibits oxidative stress in glomerular mesangial cells alleviating diabetic nephropathy by regulating SIRT3/FOXO1 signaling. Kidney Blood Press Res. (2023) 48:738–51. doi: 10.1159/000534832, PMID: 37935137

[48] Zhao Y Shao C Zhou H Yu L Bao Y Mao Q . Salvianolic acid B inhibits atherosclerosis and TNF-alpha-induced inflammation by regulating NF-kappaB/NLRP3 signaling pathway. Phytomedicine. (2023) 119:155002. doi: 10.1016/j.phymed.2023.155002, PMID: 37572566

[49] Guan Y Li L Kan L Xie Q . Inhalation of salvianolic acid B prevents fine particulate matter-induced acute airway inflammation and oxidative stress by downregulating the LTR4/myD88/NLRP3 pathway. Oxid Med Cell Longev. (2022) 2022:5044356. doi: 10.1155/2022/5044356, PMID: 35795853 PMC9252752

[50] Zhang Y Feng X Du M Ding J Liu P . Salvianolic acid B attenuates the inflammatory response in atherosclerosis by regulating MAPKs/ NF-kappaB signaling pathways in LDLR-/- mice and RAW264.7 cells. Int J Immunopathol Pharmacol. (2022) 36:3946320221079468. doi: 10.1177/03946320221079468, PMID: 35285334 PMC9118216

[51] Wang T Wang J Xu H Yan H Liu Y Zhang N . Salvianolic acid B alleviates autoimmunity in Treg-deficient mice via inhibiting IL2-STAT5 signaling. Phytother Res. (2024) 38:3825–36. doi: 10.1002/ptr.8222, PMID: 38887974

[52] Wang Y Chen G Yu X Li Y Zhang L He Z . Salvianolic acid B ameliorates cerebral ischemia/reperfusion injury through inhibiting TLR4/myD88 signaling pathway. Inflammation. (2016) 39:1503–13. doi: 10.1007/s10753-016-0384-5, PMID: 27255374

[53] Zhang ML Zhang MN Chen H Wang X Zhao K Li X . Salvianolic acid B alleviates high glucose-induced vascular smooth muscle cell inflammation by upregulating the miR-486a-5p expression. Mediators Inflammation. (2024) 2024:4121166. doi: 10.1155/2024/4121166, PMID: 38405620 PMC10890902

[54] Zou T Gao S Yu Z Zhang F Yao L Xu M . Salvianolic acid B inhibits RAW264.7 cell polarization towards the M1 phenotype by inhibiting NF-kappaB and Akt/mTOR pathway activation. Sci Rep. (2022) 12:13857. doi: 10.1038/s41598-022-18246-0, PMID: 35974091 PMC9381594

[55] Wang J Ma Y Guo M Yang H Guan X . Salvianolic acid B suppresses EMT and apoptosis to lessen drug resistance through AKT/mTOR in gastric cancer cells. Cytotechnology. (2021) 73:49–61. doi: 10.1007/s10616-020-00441-4, PMID: 33505113 PMC7817758

[56] Xu W Shi Z Yu X Xu Y Chen Y He Y . Salvianolic acid B exerts an anti-hepatocellular carcinoma effect by regulating the Hippo/YAP pathway and promoting pSmad3L to pSmad3C simultaneously. Eur J Pharmacol. (2023) 939:175423. doi: 10.1016/j.ejphar.2022.175423, PMID: 36509132

[57] Wei J Wu J Xu W Nie H Zhou R Wang R . Salvianolic acid B inhibits glycolysis in oral squamous cell carcinoma via targeting PI3K/AKT/HIF-1alpha signaling pathway. Cell Death Dis. (2018) 9:599. doi: 10.1038/s41419-018-0623-9, PMID: 29789538 PMC5964095

[58] Chen Y Hu M Wang S Wang Q Lu H Wang F . Nano-delivery of salvianolic acid B induces the quiescence of tumor-associated fibroblasts via interfering with TGF-beta1/Smad signaling to facilitate chemo- and immunotherapy in desmoplastic tumor. Int J Pharm. (2022) 623:121953. doi: 10.1016/j.ijpharm.2022.121953, PMID: 35753535

[59] Teng M Hu C Yang B Xiao W Zhou Q Li Y . Salvianolic acid B targets mortalin and inhibits the migration and invasion of hepatocellular carcinoma via the RECK/STAT3 pathway. Cancer Cell Int. (2021) 21:654. doi: 10.1186/s12935-021-02367-z, PMID: 34876128 PMC8650508

[60] Yang Y Huang L Gao J Qian B . Salvianolic acid B inhibits the growth and metastasis of A549 lung cancer cells through the NDRG2/PTEN pathway by inducing oxidative stress. Med Oncol. (2024) 41:170. doi: 10.1007/s12032-024-02413-6, PMID: 38847902

[61] Xu S Zhong A Ma H Li D Hu Y Xu Y . Neuroprotective effect of salvianolic acid B against cerebral ischemic injury in rats via the CD40/NF-kappaB pathway associated with suppression of platelets activation and neuroinflammation. Brain Res. (2017) 1661:37–48. doi: 10.1016/j.brainres.2017.02.011, PMID: 28214521

[62] Ma X Xu W Zhang Z Liu N Yang J Wang M . Salvianolic acid B ameliorates cognitive deficits through IGF-1/akt pathway in rats with vascular dementia. Cell Physiol Biochem. (2017) 43:1381–91. doi: 10.1159/000481849, PMID: 28992623

[63] Liu X Hou Z Han M Chen K Wang Y Qing J . Salvianolic acid B alleviates comorbid pain in depression induced by chronic restraint stress through inhibiting GABAergic neuron excitation via an ERK-CREB-BDNF axis-dependent mechanism. J Psychiatr Res. (2022) 151:205–16. doi: 10.1016/j.jpsychires.2022.04.014, PMID: 35500448

[64] Xiong Y Wang J Chu H Chen D Guo H . Salvianolic Acid B Restored Impaired Barrier Function via Downregulation of MLCK by microRNA-1 in Rat Colitis Model. Front Pharmacol. (2016) 7:134. doi: 10.3389/fphar.2016.00134, PMID: 27303297 PMC4880571

[65] Liu J Dong W Gao C Meng Y . Salvianolic acid B protects cardiomyocytes from ischemia/reperfusion injury by mediating circTRRAP/miR-214-3p/SOX6 axis. Int Heart J. (2022) 63:1176–86. doi: 10.1536/ihj.22-102, PMID: 36450557

[66] Yu J Chen R Tan Y Wu J Qi J Zhang M . Salvianolic acid B alleviates heart failure by inactivating ERK1/2/GATA4 signaling pathway after pressure overload in mice. PloS One. (2016) 11:e0166560. doi: 10.1371/journal.pone.0166560, PMID: 27893819 PMC5125602

[67] Wu Y Lin Z Bao Y Liu Y Zhang X . Salvianolic acid B ameliorated chemotherapeutic injury of cardiac myocytes through the nrf2/ARE signaling pathway. Discov Med. (2024) 36:415–23. doi: 10.24976/Discov.Med.202436181.39, PMID: 38409846

[68] Liu H Liu W Qiu H Zou D Cai H Chen Q . Salvianolic acid B protects against myocardial ischaemia-reperfusion injury in rats via inhibiting high mobility group box 1 protein expression through the PI3K/Akt signalling pathway. Naunyn Schmiedebergs Arch Pharmacol. (2020) 393:1527–39. doi: 10.1007/s00210-019-01755-7, PMID: 31853618 PMC7351826

[69] Wang J Zhang Y Guo LL Wu GJ Liu RH . Salvianolic acid B inhibits the TLR4-NFkappaB-TNFalpha pathway and attenuates neonatal rat cardiomyocyte injury induced by lipopolysaccharide. Chin J Integr Med. (2011) 17:775–9. doi: 10.1007/s11655-011-0877-x, PMID: 22101700

[70] Wang YM Chu TJ Wan RT Niu WP Bian YF Li J . Quercetin ameliorates atherosclerosis by inhibiting inflammation of vascular endothelial cells via Piezo1 channels. Phytomedicine. (2024) 132:155865. doi: 10.1016/j.phymed.2024.155865, PMID: 39004029

[71] Jia M Li Q Guo J Shi W Zhu L Huang Y . Deletion of BACH1 attenuates atherosclerosis by reducing endothelial inflammation. Circ Res. (2022) 130:1038–55. doi: 10.1161/CIRCRESAHA.121.319540, PMID: 35196865

[72] Bu LL Yuan HH Xie LL Guo MH Liao DF Zheng XL . New dawn for atherosclerosis: vascular endothelial cell senescence and death. Int J Mol Sci. (2023) 24:15160. doi: 10.3390/ijms242015160, PMID: 37894840 PMC10606899

[73] Chen YH Lin SJ Ku HH Shiao MS Lin FY Chen JW . Salvianolic acid B attenuates VCAM-1 and ICAM-1 expression in TNF-alpha-treated human aortic endothelial cells. J Cell Biochem. (2001) 82:512–21. doi: 10.1002/jcb.1176, PMID: 11500927

[74] Zhou Z Liu Y Miao AD Wang SQ . Salvianolic acid B attenuates plasminogen activator inhibitor type 1 production in TNF-alpha treated human umbilical vein endothelial cells. J Cell Biochem. (2005) 96:109–16. doi: 10.1002/jcb.20567, PMID: 16052513

[75] Xu S Zhong A Bu X Ma H Li W Xu X . Salvianolic acid B inhibits platelets-mediated inflammatory response in vascular endothelial cells. Thromb Res. (2015) 135:137–45. doi: 10.1016/j.thromres.2014.10.034, PMID: 25466843

[76] Qui Y Rui YC Zhang L Li TJ Zhang WD . VEGF induced hyperpermeability in bovine aortic endothelial cell and inhibitory effect of salvianolic acid B. Acta Pharmacol Sin. (2001) 22:117–20., PMID: 12545975

[77] Ding M Ye TX Zhao GR Yuan YJ Guo ZX . Aqueous extract of Salvia miltiorrhiza attenuates increased endothelial permeability induced by tumor necrosis factor-alpha. Int Immunopharmacol. (2005) 5:1641–51. doi: 10.1016/j.intimp.2005.05.005, PMID: 16039554

[78] Ba J Peng H Chen Y Gao Y . Effects and mechanism analysis of vascular endothelial growth factor and salvianolic acid B on 125I-low density lipoprotein permeability of the rabbit aortary endothelial cells. Cell Biochem Biophys. (2014) 70:1533–8. doi: 10.1007/s12013-014-0089-z, PMID: 25005771

[79] Joe Y Zheng M Kim HJ Kim S Uddin MJ Park C . Salvianolic acid B exerts vasoprotective effects through the modulation of heme oxygenase-1 and arginase activities. J Pharmacol Exp Ther. (2012) 341:850–8. doi: 10.1124/jpet.111.190736, PMID: 22442118

[80] Pan X Wan R Wang Y Liu S He Y Deng B . Inhibition of chemically and mechanically activated Piezo1 channels as a mechanism for ameliorating atherosclerosis with salvianolic acid B. Br J Pharmacol. (2022) 179:3778–814. doi: 10.1111/bph.15826, PMID: 35194776

[81] Tang Y Wa Q Peng L Zheng Y Chen J Chen X . Salvianolic acid B suppresses ER stress-induced NLRP3 inflammasome and pyroptosis via the AMPK/foxO4 and syndecan-4/rac1 signaling pathways in human endothelial progenitor cells. Oxid Med Cell Longev. (2022) 2022:8332825. doi: 10.1155/2022/8332825, PMID: 35340217 PMC8947883

[82] Chen YL Hu CS Lin FY Chen YH Sheu LM Ku HH . Salvianolic acid B attenuates cyclooxygenase-2 expression *in vitro* in LPS-treated human aortic smooth muscle cells and *in vivo* in the apolipoprotein-E-deficient mouse aorta. J Cell Biochem. (2006) 98:618–31. doi: 10.1002/jcb.20793, PMID: 16440326

[83] Yang TL Lin FY Chen YH Chiu JJ Shiao MS Tsai CS . Salvianolic acid B inhibits low-density lipoprotein oxidation and neointimal hyperplasia in endothelium-denuded hypercholesterolaemic rabbits. J Sci Food Agric. (2011) 91:134–41. doi: 10.1002/jsfa.4163, PMID: 20824680

[84] Chen SC Lin YL Huang B Wang DL Cheng JJ . Salvianolic acid B suppresses IFN-gamma-induced JAK/STAT1 activation in endothelial cells. Thromb Res. (2011) 128:560–4. doi: 10.1016/j.thromres.2011.08.032, PMID: 21992896

[85] Demers A Samami S Lauzier B Des Rosiers C Ngo Sock ET Ong H . PCSK9 induces CD36 degradation and affects long-chain fatty acid uptake and triglyceride metabolism in adipocytes and in mouse liver. Arterioscler Thromb Vasc Biol. (2015) 35:2517–25. doi: 10.1161/ATVBAHA.115.306032, PMID: 26494228

[86] Li Z Zou X Lu R Wan X Sun S Wang S . Arsenic trioxide alleviates atherosclerosis by inhibiting CD36-induced endocytosis and TLR4/NF-kappaB-induced inflammation in macrophage and ApoE(-/-) mice. Int Immunopharmacol. (2024) 128:111452. doi: 10.1016/j.intimp.2023.111452, PMID: 38237221

[87] Silverstein RL . Inflammation, atherosclerosis, and arterial thrombosis: role of the scavenger receptor CD36. Cleve Clin J Med. (2009) 76:S27–30. doi: 10.3949/ccjm.76.s2.06, PMID: 19376978 PMC2810530

[88] Wang L Bao Y Yang Y Wu Y Chen X Si S . Discovery of antagonists for human scavenger receptor CD36 via an ELISA-like high-throughput screening assay. J Biomol Screen. (2010) 15:239–50. doi: 10.1177/1087057109359686, PMID: 20150587

[89] Bao Y Wang L Xu Y Yang Y Wang L Si S . Salvianolic acid B inhibits macrophage uptake of modified low density lipoprotein (mLDL) in a scavenger receptor CD36-dependent manner. Atherosclerosis. (2012) 223:152–9. doi: 10.1016/j.atherosclerosis.2012.05.006, PMID: 22658257 PMC3389144

[90] Sun A Liu H Wang S Shi D Xu L Cheng Y . Salvianolic acid B suppresses maturation of human monocyte-derived dendritic cells by activating PPARgamma. Br J Pharmacol. (2011) 164:2042–53. doi: 10.1111/j.1476-5381.2011.01518.x, PMID: 21649636 PMC3246666

[91] Sun M Ye Y Huang Y Yin W Yu Z Wang S . Salvianolic acid B improves autophagic dysfunction and decreases the apoptosis of cholesterol crystal−induced macrophages via inhibiting the Akt/mTOR signaling pathway. Mol Med Rep. (2021) 24:763. doi: 10.3892/mmr.2021.12403, PMID: 34490483 PMC8430306

[92] Yang Y Pei K Zhang Q Wang D Feng H Du Z . Salvianolic acid B ameliorates atherosclerosis via inhibiting YAP/TAZ/JNK signaling pathway in endothelial cells and pericytes. Biochim Biophys Acta Mol Cell Biol Lipids. (2020) 1865:158779. doi: 10.1016/j.bbalip.2020.158779, PMID: 32739616

[93] He H Shi M Yang X Zeng X Wu L Li L . Comparison of cardioprotective effects using salvianolic acid B and benazepril for the treatment of chronic myocardial infarction in rats. Naunyn Schmiedebergs Arch Pharmacol. (2008) 378:311–22. doi: 10.1007/s00210-008-0287-6, PMID: 18500511

[94] Tan JZ Li QW Nan YY . Effects of salvianolic acid B preconditioning endothelial progenitor cells on expressions of myocardial genes in bone mesenchymal stem cells at the early cell differentiation stage of rats. Zhongguo Zhong Xi Yi Jie He Za Zhi. (2009) 29:529–32., PMID: 19702086

[95] Jiang B Chen J Xu L Gao Z Deng Y Wang Y . Salvianolic acid B functioned as a competitive inhibitor of matrix metalloproteinase-9 and efficiently prevented cardiac remodeling. BMC Pharmacol. (2010) 10:10. doi: 10.1186/1471-2210-10-10, PMID: 20735854 PMC2940789

[96] Zhao GF Fan YC Jiang XJ . Effects of the proliferation state of the endothelial progenitor cells preconditioned with salvianolic acid B and bone marrow mesenchymal stem cells transplanted in acute myocardial infarction rats. Zhongguo Zhong Xi Yi Jie He Za Zhi. (2012) 32:671–5., PMID: 22679732

[97] Guo HD Cui GH Tian JX Lu PP Zhu QC Lv R . Transplantation of salvianolic acid B pretreated mesenchymal stem cells improves cardiac function in rats with myocardial infarction through angiogenesis and paracrine mechanisms. Int J Cardiol. (2014) 177:538–42. doi: 10.1016/j.ijcard.2014.08.104, PMID: 25189503

[98] Pan C Lou L Huo Y Singh G Chen M Zhang D . Salvianolic acid B and tanshinone IIA attenuate myocardial ischemia injury in mice by NO production through multiple pathways. Ther Adv Cardiovasc Dis. (2011) 5:99–111. doi: 10.1177/1753944710396538, PMID: 21282198

[99] Lin C Liu Z Lu Y Yao Y Zhang Y Ma Z . Cardioprotective effect of Salvianolic acid B on acute myocardial infarction by promoting autophagy and neovascularization and inhibiting apoptosis. J Pharm Pharmacol. (2016) 68:941–52. doi: 10.1111/jphp.12567, PMID: 27139338

[100] Xu L Deng Y Feng L Li D Chen X Ma C . Cardio-protection of salvianolic acid B through inhibition of apoptosis network. PloS One. (2011) 6:e24036. doi: 10.1371/journal.pone.0024036, PMID: 21915278 PMC3167815

[101] Chen Q Xu Q Zhu H Wang J Sun N Bian H . Salvianolic acid B promotes angiogenesis and inhibits cardiomyocyte apoptosis by regulating autophagy in myocardial ischemia. Chin Med. (2023) 18:155. doi: 10.1186/s13020-023-00859-w, PMID: 38017536 PMC10685573

[102] Li Q Zuo Z Pan Y Zhang Q Xu L Jiang B . Salvianolic acid B alleviates myocardial ischemia injury by suppressing NLRP3 inflammasome activation via SIRT1-AMPK-PGC-1alpha signaling pathway. Cardiovasc Toxicol. (2022) 22:842–57. doi: 10.1007/s12012-022-09760-8, PMID: 35809215

[103] Shen Y Shen X Wang S Zhang Y Wang Y Ding Y . Protective effects of Salvianolic acid B on rat ferroptosis in myocardial infarction through upregulating the Nrf2 signaling pathway. Int Immunopharmacol. (2022) 112:109257. doi: 10.1016/j.intimp.2022.109257, PMID: 36174419

[104] Deng Y Zhang T Teng F Li D Xu F Cho K . Ginsenoside Rg1 and Rb1, in combination with salvianolic acid B, play different roles in myocardial infarction in rats. J Chin Med Assoc. (2015) 78:114–20. doi: 10.1016/j.jcma.2014.10.001, PMID: 25476150

[105] Qiu J Cai G Liu X Ma D . alpha(v)beta(3) integrin receptor specific peptide modified, salvianolic acid B and panax notoginsenoside loaded nanomedicine for the combination therapy of acute myocardial ischemia. BioMed Pharmacother. (2017) 96:1418–26. doi: 10.1016/j.biopha.2017.10.086, PMID: 29079344

[106] Shoba E Lakra R Kiran MS Korrapati PS . Strategic design of cardiac mimetic core-shell nanofibrous scaffold impregnated with Salvianolic acid B and Magnesium l-ascorbic acid 2 phosphate for myoblast differentiation. Mater Sci Eng C Mater Biol Appl. (2018) 90:131–47. doi: 10.1016/j.msec.2018.04.056, PMID: 29853076

[107] Chen R Zhu C Xu L Gu Y Ren S Bai H . An injectable peptide hydrogel with excellent self-healing ability to continuously release salvianolic acid B for myocardial infarction. Biomaterials. (2021) 274:120855. doi: 10.1016/j.biomaterials.2021.120855, PMID: 33975276

[108] Qiao Z Ma J Liu H . Evaluation of the antioxidant potential of Salvia miltiorrhiza ethanol extract in a rat model of ischemia-reperfusion injury. Molecules. (2011) 16:10002–12. doi: 10.3390/molecules161210002, PMID: 22138858 PMC6264289

[109] Qiao Z Xu Y . Salvianolic acid B alleviating myocardium injury in ischemia reperfusion rats. Afr J Tradit Complement Altern Med. (2016) 13:157–61. doi: 10.21010/ajtcam.v13i4.20, PMID: 28852731 PMC5566139

[110] Gao F Sun G Ren X Nie Y Sun J Qin M . Protective effect of salvianolic acid B on isolated heart ischemia/reperfusion injury in rats. Zhongguo Zhong Yao Za Zhi. (2012) 37:358–61., PMID: 22568240

[111] Xue L Wu Z Ji XP Gao XQ Guo YH . Effect and mechanism of salvianolic acid B on the myocardial ischemia-reperfusion injury in rats. Asian Pac J Trop Med. (2014) 7:280–4. doi: 10.1016/S1995-7645(14)60038-9, PMID: 24507676

[112] Zhao M Li F Jian Y Wang X Yang H Wang J . Salvianolic acid B regulates macrophage polarization in ischemic/reperfused hearts by inhibiting mTORC1-induced glycolysis. Eur J Pharmacol. (2020) 871:172916. doi: 10.1016/j.ejphar.2020.172916, PMID: 31930970

[113] Li D Wang J Hou J Fu J Liu J Lin R . Salvianolic acid B induced upregulation of miR-30a protects cardiac myocytes from ischemia/reperfusion injury. BMC Complement Altern Med. (2016) 16:336. doi: 10.1186/s12906-016-1275-x, PMID: 27586425 PMC5009695

[114] Mao Q Shao C Zhou H Yu L Bao Y Zhao Y . Exploring the mechanism of salvianolic acid B against myocardial ischemia-reperfusion injury based on network pharmacology. Pharm (Basel). (2024) 17:309. doi: 10.3390/ph17030309, PMID: 38543095 PMC10974641

[115] Deng Y Yang M Xu F Zhang Q Zhao Q Yu H . Combined salvianolic acid B and ginsenoside rg1 exerts cardioprotection against ischemia/reperfusion injury in rats. PloS One. (2015) 10:e0135435. doi: 10.1371/journal.pone.0135435, PMID: 26280455 PMC4539231

[116] Liu M Ye J Gao S Fang W Li H Geng B . Salvianolic acid B protects cardiomyocytes from angiotensin II-induced hypertrophy via inhibition of PARP-1. Biochem Biophys Res Commun. (2014) 444:346–53. doi: 10.1016/j.bbrc.2014.01.045, PMID: 24462865

[117] Ma D Mandour AS Yoshida T Matsuura K Shimada K Kitpipatkun P . Intraventricular pressure gradients change during the development of left ventricular hypertrophy: Effect of salvianolic acid B and beta-blocker. Ultrasound. (2021) 29:229–40. doi: 10.1177/1742271X20987584, PMID: 34777543 PMC8579373

[118] Dillmann WH . Diabetic cardiomyopathy. Circ Res. (2019) 124:1160–2. doi: 10.1161/CIRCRESAHA.118.314665, PMID: 30973809 PMC6578576

[119] Zhao X Liu S Wang X Chen Y Pang P Yang Q . Diabetic cardiomyopathy: Clinical phenotype and practice. Front Endocrinol (Lausanne). (2022) 13:1032268. doi: 10.3389/fendo.2022.1032268, PMID: 36568097 PMC9767955

[120] Jia G Whaley-Connell A Sowers JR . Diabetic cardiomyopathy: a hyperglycaemia- and insulin-resistance-induced heart disease. Diabetologia. (2018) 61:21–8. doi: 10.1007/s00125-017-4390-4, PMID: 28776083 PMC5720913

[121] Li CL Liu B Wang ZY Xie F Qiao W Cheng J . Salvianolic acid B improves myocardial function in diabetic cardiomyopathy by suppressing IGFBP3. J Mol Cell Cardiol. (2020) 139:98–112. doi: 10.1016/j.yjmcc.2020.01.009, PMID: 31982427

[122] Luo H Fu L Wang X Yini X Ling T Shen X . Salvianolic acid B ameliorates myocardial fibrosis in diabetic cardiomyopathy by deubiquitinating Smad7. Chin Med. (2023) 18:161. doi: 10.1186/s13020-023-00868-9, PMID: 38072948 PMC10712074

[123] Beesley SJ Weber G Sarge T Nikravan S Grissom CK Lanspa MJ . Septic cardiomyopathy. Crit Care Med. (2018) 46:625–34. doi: 10.1097/CCM.0000000000002851, PMID: 29227368

[124] Wang R Xu Y Fang Y Wang C Xue Y Wang F . Pathogenetic mechanisms of septic cardiomyopathy. J Cell Physiol. (2022) 237:49–58. doi: 10.1002/jcp.30527, PMID: 34278573

[125] Hollenberg SM Singer M . Pathophysiology of sepsis-induced cardiomyopathy. Nat Rev Cardiol. (2021) 18:424–34. doi: 10.1038/s41569-020-00492-2, PMID: 33473203

[126] Chen R Zheng A Wang Y Guo L Dou H Lu L . Salvianolic acid B improves mitochondrial dysfunction of septic cardiomyopathy via enhancing ATF5-mediated mitochondrial unfolded protein response. Toxicol Appl Pharmacol. (2024) 491:117072. doi: 10.1016/j.taap.2024.117072, PMID: 39153513

[127] D'Agostino M Mauro D Zicarelli M Carullo N Greco M Andreucci M . miRNAs in uremic cardiomyopathy: A comprehensive review. Int J Mol Sci. (2023) 24:5425. doi: 10.3390/ijms24065425, PMID: 36982497 PMC10049249

[128] Hiraiwa H Kasugai D Okumura T Murohara T . Implications of uremic cardiomyopathy for the practicing clinician: an educational review. Heart Fail Rev. (2023) 28:1129–39. doi: 10.1007/s10741-023-10318-1, PMID: 37173614 PMC10403419

[129] Nguyen TD Schulze PC . Cardiac metabolism in heart failure and implications for uremic cardiomyopathy. Circ Res. (2023) 132:1034–49. doi: 10.1161/CIRCRESAHA.123.321759, PMID: 37053280

[130] Ma D Mandour AS Elfadadny A Hendawy H Yoshida T El-Husseiny HM . Changes in cardiac function during the development of uremic cardiomyopathy and the effect of salvianolic acid B administration in a rat model. Front Vet Sci. (2022) 9:905759. doi: 10.3389/fvets.2022.905759, PMID: 35782566 PMC9244798

[131] Shi S Chen Y Luo Z Nie G Dai Y . Role of oxidative stress and inflammation-related signaling pathways in doxorubicin-induced cardiomyopathy. Cell Commun Signal. (2023) 21:61. doi: 10.1186/s12964-023-01077-5, PMID: 36918950 PMC10012797

[132] Glembotski CC Bagchi S Blackwood EA . ER-specific autophagy or ER-phagy in cardiac myocytes protects the heart against doxorubicin-induced cardiotoxicity. JACC CardioOncol. (2023) 5:671–3. doi: 10.1016/j.jaccao.2023.08.004, PMID: 37969647 PMC10635872

[133] Malik A Bagchi AK Jassal DS Singal PK . Interleukin-10 mitigates doxorubicin-induced endoplasmic reticulum stress as well as cardiomyopathy. Biomedicines. (2022) 10:890. doi: 10.3390/biomedicines10040890, PMID: 35453640 PMC9027958

[134] Chen R Sun G Yang L Wang J Sun X . Salvianolic acid B protects against doxorubicin induced cardiac dysfunction via inhibition of ER stress mediated cardiomyocyte apoptosis. Toxicol Res (Camb). (2016) 5:1335–45. doi: 10.1039/c6tx00111d, PMID: 30090438 PMC6062089

[135] Chen RC Sun GB Ye JX Wang J Zhang MD Sun XB . Salvianolic acid B attenuates doxorubicin-induced ER stress by inhibiting TRPC3 and TRPC6 mediated Ca(2+) overload in rat cardiomyocytes. Toxicol Lett. (2017) 276:21–30. doi: 10.1016/j.toxlet.2017.04.010, PMID: 28495616

[136] Jia Y Guo H Cheng X Zhang Y Si M Shi J . Hesperidin protects against cisplatin-induced cardiotoxicity in mice by regulating the p62-Keap1-Nrf2 pathway. Food Funct. (2022) 13:4205–15. doi: 10.1039/d2fo00298a, PMID: 35332348

[137] Xia J Hu JN Zhang RB Liu W Zhang H Wang Z . Icariin exhibits protective effects on cisplatin-induced cardiotoxicity via ROS-mediated oxidative stress injury *in vivo* and *in vitro*. Phytomedicine. (2022) 104:154331. doi: 10.1016/j.phymed.2022.154331, PMID: 35878553

[138] Xing JJ Mi XJ Hou JG Cai EB Zheng SW Wang SH . Maltol mitigates cisplatin-evoked cardiotoxicity via inhibiting the PI3K/Akt signaling pathway in rodents *in vivo* and *in vitro*. Phytother Res. (2022) 36:1724–35. doi: 10.1002/ptr.7405, PMID: 35174550

[139] Lin Z Bao Y Hong B Wang Y Zhang X Wu Y . Salvianolic acid B attenuated cisplatin-induced cardiac injury and oxidative stress via modulating Nrf2 signal pathway. J Toxicol Sci. (2021) 46:199–207. doi: 10.2131/jts.46.199, PMID: 33952797

[140] Akbar N Anum H Razzaq SS Salim A Usman S Haneef K . Ascorbic acid and salvianolic acid B enhance the valproic acid and 5-azacytidinemediated cardiac differentiation of mesenchymal stem cells. Mol Biol Rep. (2023) 50:7371–80. doi: 10.1007/s11033-023-08634-8, PMID: 37450078

[141] Kong XL Lyu Q Zhang YQ Kang DF Li C Zhang L . Effect of astragaloside IV and salvianolic acid B on antioxidant stress and vascular endothelial protection in the treatment of atherosclerosis based on metabonomics. Chin J Nat Med. (2022) 20:601–13. doi: 10.1016/S1875-5364(22)60186-9, PMID: 36031232

[142] Yang K Luo Y Lu S Hu R Du Y Liao P . Salvianolic acid B and ginsenoside re synergistically protect against ox-LDL-induced endothelial apoptosis through the antioxidative and antiinflammatory mechanisms. Front Pharmacol. (2018) 9:662. doi: 10.3389/fphar.2018.00662, PMID: 29973885 PMC6019702

[143] Li Y Wang L Dong Z Wang S Qi L Cho K . Cardioprotection of salvianolic acid B and ginsenoside Rg1 combination on subacute myocardial infarction and the underlying mechanism. Phytomedicine. (2019) 57:255–61. doi: 10.1016/j.phymed.2018.12.040, PMID: 30797987

[144] Shen H Zhang Y Shao Y Chen S Yin P Liu X . Synergism of salvianolic acid B and ginsenoside Rg1 magnifies the therapeutic potency against ischemic stroke. Neuroreport. (2024) 35:1041–51. doi: 10.1097/WNR.0000000000002099, PMID: 39292959 PMC11424057

[145] Fu Y Xing R Wang L Yang L Jiang B . Neurovascular protection of salvianolic acid B and ginsenoside Rg1 combination against acute ischemic stroke in rats. Neuroreport. (2021) 32:1140–6. doi: 10.1097/WNR.0000000000001706, PMID: 34284451

[146] Zhao Q Yang M Deng Y Yu H Wang L Teng F . The safety evaluation of salvianolic acid B and ginsenoside rg1 combination on mice. Int J Mol Sci. (2015) 16:29345–56. doi: 10.3390/ijms161226176, PMID: 26690140 PMC4691119

[147] Zhang Q Li Y Wang S Gu D Zhang C Xu S . Chitosan-based oral nanoparticles as an efficient platform for kidney-targeted drug delivery in the treatment of renal fibrosis. Int J Biol Macromol. (2024) 256:128315. doi: 10.1016/j.ijbiomac.2023.128315, PMID: 38000609

[148] Li J Zhang C He W Qiao H Chen J Wang K . Coordination-driven assembly of catechol-modified chitosan for the kidney-specific delivery of salvianolic acid B to treat renal fibrosis. Biomater Sci. (2017) 6:179–88. doi: 10.1039/c7bm00811b, PMID: 29170782

[149] Grossi C Guccione C Isacchi B Bergonzi MC Luccarini I Casamenti F . Development of blood-brain barrier permeable nanoparticles as potential carriers for salvianolic acid B to CNS. Planta Med. (2017) 83:382–91. doi: 10.1055/s-0042-101945, PMID: 27002395

[150] Isacchi B Fabbri V Galeotti N Bergonzi MC Karioti A Ghelardini C . Salvianolic acid B and its liposomal formulations: anti-hyperalgesic activity in the treatment of neuropathic pain. Eur J Pharm Sci. (2011) 44:552–8. doi: 10.1016/j.ejps.2011.09.019, PMID: 22001125

[151] Shi J Guo S Wu Y Chen G Lai J Xu X . Behaviour of cell penetrating peptide TAT-modified liposomes loaded with salvianolic acid B on the migration, proliferation, and survival of human skin fibroblasts. J Liposome Res. (2020) 30:93–106. doi: 10.1080/08982104.2019.1593451, PMID: 31012367

[152] Lihao Q Tingting L Jiawei Z Yifei B Zheyu T Jingyan L . 3D bioprinting of Salvianolic acid B-sodium alginate-gelatin skin scaffolds promotes diabetic wound repair via antioxidant, anti-inflammatory, and proangiogenic effects. BioMed Pharmacother. (2024) 171:116168. doi: 10.1016/j.biopha.2024.116168, PMID: 38232662

[153] Li J Wang Q Zhi W Wang J Feng B Qu S . Immobilization of salvianolic acid B-loaded chitosan microspheres distributed three-dimensionally and homogeneously on the porous surface of hydroxyapatite scaffolds. BioMed Mater. (2016) 11:55014. doi: 10.1088/1748-6041/11/5/055014, PMID: 27716647

[154] Liu S Xu Z Hu J Wu Z Zheng Y . Preparation and sustained-release properties of poly(lactic acid)/graphene oxide porous biomimetic composite scaffolds loaded with salvianolic acid B. RSC Adv. (2022) 12:28867–77. doi: 10.1039/d2ra05371c, PMID: 36329763 PMC9585927

[155] Zhou G Cao Y Yan Y Xu H Zhang X Yan T . Injectable hydrogels based on hyaluronic acid and gelatin combined with salvianolic acid B and vascular endothelial growth factor for treatment of traumatic brain injury in mice. Molecules. (2024) 29:1705. doi: 10.3390/molecules29081705, PMID: 38675525 PMC11052029

[156] Wu L Wei Z He S Bi Y Cao Y Wang W . Mesoporous bioactive glass scaffold delivers salvianolic acid B to promote bone regeneration in a rat cranial defect model. Curr Drug Delivery. (2021) 18:323–33. doi: 10.2174/1567201817666200916091253, PMID: 32938350

[157] Kan SL Li J Liu JP Zhao Y . Preparation and IVIVC evaluation of salvianolic acid B micro-porous osmotic pump pellets. Drug Dev Ind Pharm. (2015) 41:476–81. doi: 10.3109/03639045.2013.879722, PMID: 24467406

[158] Meng LC Zheng JY Qiu YH Zheng L Zheng JY Liu YQ . Salvianolic acid B ameliorates non-alcoholic fatty liver disease by inhibiting hepatic lipid accumulation and NLRP3 inflammasome in ob/ob mice. Int Immunopharmacol. (2022) 111:109099. doi: 10.1016/j.intimp.2022.109099, PMID: 35932615

[159] Zhang N Hu Y Ding C Zeng W Shan W Fan H . Salvianolic acid B protects against chronic alcoholic liver injury via SIRT1-mediated inhibition of CRP and ChREBP in rats. Toxicol Lett. (2017) 267:1–10. doi: 10.1016/j.toxlet.2016.12.010, PMID: 27989594

[160] Zeng W Shan W Gao L Gao D Hu Y Wang G . Inhibition of HMGB1 release via salvianolic acid B-mediated SIRT1 up-regulation protects rats against non-alcoholic fatty liver disease. Sci Rep. (2015) 5:16013. doi: 10.1038/srep16013, PMID: 26525891 PMC4630617

[161] Lv H Wang L Shen J Hao S Ming A Wang X . Salvianolic acid B attenuates apoptosis and inflammation via SIRT1 activation in experimental stroke rats. Brain Res Bull. (2015) 115:30–6. doi: 10.1016/j.brainresbull.2015.05.002, PMID: 25981395

[162] Chen T Liu W Chao X Zhang L Qu Y Huo J . Salvianolic acid B attenuates brain damage and inflammation after traumatic brain injury in mice. Brain Res Bull. (2011) 84:163–8. doi: 10.1016/j.brainresbull.2010.11.015, PMID: 21134421

[163] Zhang X Wu Q Lu Y Wan J Dai H Zhou X . Cerebroprotection by salvianolic acid B after experimental subarachnoid hemorrhage occurs via Nrf2- and SIRT1-dependent pathways. Free Radic Biol Med. (2018) 124:504–16. doi: 10.1016/j.freeradbiomed.2018.06.035, PMID: 29966698 PMC6286712

[164] Tongqiang L Shaopeng L Xiaofang Y Nana S Xialian X Jiachang H . Salvianolic acid B prevents iodinated contrast media-induced acute renal injury in rats via the PI3K/akt/nrf2 pathway. Oxid Med Cell Longev. (2016) 2016:7079487. doi: 10.1155/2016/7079487, PMID: 27382429 PMC4921628

[165] Wang Y Chang J Qiao S Yang Y Yun C Li Y . Salvianolic acid B attenuates diabetic nephropathy through alleviating ADORA2B, NALP3 in flammasome, and NFkappaB activity. Can J Physiol Pharmacol. (2024) 102:318–30. doi: 10.1139/cjpp-2023-0089, PMID: 38070193

[166] Chen J Hu Q Luo Y Luo L Lin H Chen D . Salvianolic acid B attenuates membranous nephropathy by activating renal autophagy via microRNA-145-5p/phosphatidylinositol 3-kinase/AKT pathway. Bioengineered. (2022) 13:13956–69. doi: 10.1080/21655979.2022.2083822, PMID: 35723058 PMC9345616

[167] Li Y Chen R Wu J Xue X Liu T Peng G . Salvianolic acid B protects against pulmonary fibrosis by attenuating stimulating protein 1-mediated macrophage and alveolar type 2 cell senescence. Phytother Res. (2024) 38:620–35. doi: 10.1002/ptr.8070, PMID: 37953063

[168] Yang CW Liu H Li XD Sui SG Liu YF . Salvianolic acid B protects against acute lung injury by decreasing TRPM6 and TRPM7 expressions in a rat model of sepsis. J Cell Biochem. (2018) 119:701–11. doi: 10.1002/jcb.26233, PMID: 28636082

[169] Zhang T Liu M Gao Y Li H Song L Hou H . Salvianolic acid B inhalation solution enhances antifibrotic and anticoagulant effects in a rat model of pulmonary fibrosis. BioMed Pharmacother. (2021) 138:111475. doi: 10.1016/j.biopha.2021.111475, PMID: 33774314

[170] Cheng J Long J Zhang J Han L Hu Y Liu J . Safety, tolerance, and pharmacokinetics of salvianolic acid B in healthy Chinese volunteers: A randomized, double-blind, placebo-controlled phase 1 clinical trial. Front Pharmacol. (2023) 14:1146309. doi: 10.3389/fphar.2023.1146309, PMID: 37124221 PMC10133543

[171] Jiang XW Zhang Y Yang SK Zhang H Lu K Sun GL . Efficacy of salvianolic acid B combined with triamcinolone acetonide in the treatment of oral submucous fibrosis. Oral Surg Oral Med Oral Pathol Oral Radiol. (2013) 115:339–44. doi: 10.1016/j.oooo.2012.10.006, PMID: 23260769

[172] Liu P Hu YY Liu C Zhu DY Xue HM Xu ZQ . Clinical observation of salvianolic acid B in treatment of liver fibrosis in chronic hepatitis B. World J Gastroenterol. (2002) 8:679–85. doi: 10.3748/wjg.v8.i4.679, PMID: 12174378 PMC4656320

[173] Yang J Zhang G Tian J Li C Jiang W Xing Y . Cardioprotective effect of SMND-309, a novel derivate of salvianolic acid B on acute myocardial infarction in rats. Basic Clin Pharmacol Toxicol. (2010) 106:317–23. doi: 10.1111/j.1742-7843.2009.00490.x, PMID: 19912162

[174] Mu L Dong R Li C Chen J Huang Y Li T . ROS responsive conductive microspheres loaded with salvianolic acid B as adipose derived stem cell carriers for acute myocardial infarction treatment. Biomaterials. (2025) 314:122849. doi: 10.1016/j.biomaterials.2024.122849, PMID: 39357150

[175] Wei XH Chen J Wu XF Zhang Q Xia GY Chu XY . Salvianolic acid B alleviated myocardial ischemia-reperfusion injury via modulating SIRT3-mediated crosstalk between mitochondrial ROS and NLRP3. Phytomedicine. (2025) 136:156260. doi: 10.1016/j.phymed.2024.156260, PMID: 39579610

[176] Ling WC Liu J Lau CW Murugan DD Mustafa MR Huang Y . Treatment with salvianolic acid B restores endothelial function in angiotensin II-induced hypertensive mice. Biochem Pharmacol. (2017) 136:76–85. doi: 10.1016/j.bcp.2017.04.007, PMID: 28396195

[177] Zhao X Jia H Yang S Liu Y Deng B Xu X . Salvianolic Acid B reducing portal hypertension depends on macrophages in isolated portal perfused rat livers with chronic hepatitis. Evid Based Complement Alternat Med. (2012) 2012:786365. doi: 10.1155/2012/786365, PMID: 23118797 PMC3480689

[178] Xu H Zhou Y Lu C Ping J Xu LM . Salvianolic acid B lowers portal pressure in cirrhotic rats and attenuates contraction of rat hepatic stellate cells by inhibiting RhoA signaling pathway. Lab Invest. (2012) 92:1738–48. doi: 10.1038/labinvest.2012.113, PMID: 22986787

[179] Lin SJ Lee IT Chen YH Lin FY Sheu LM Ku HH . Salvianolic acid B attenuates MMP-2 and MMP-9 expression *in vivo* in apolipoprotein-E-deficient mouse aorta and *in vitro* in LPS-treated human aortic smooth muscle cells. J Cell Biochem. (2007) 100:372–84. doi: 10.1002/jcb.21042, PMID: 16924668

